# Efficacy and Limitations of an Improved Vaccine Derived from an Updated Vaccine Strain Against H5 High Pathogenicity Avian Influenza

**DOI:** 10.3390/vaccines14040291

**Published:** 2026-03-24

**Authors:** Bao Linh Nguyen, Norikazu Isoda, Yik Lim Hew, Loc Tan Huynh, Kien Trung Le, Yo Shimazu, Daiki Kobayashi, Dang Hoang Nguyen, Tho Dang Nguyen, Duc-Huy Chu, Diep Thi Nguyen, Koki Takeichi, Yuto Nanba, Takahiro Hiono, Takashi Sasaki, Yoshihiro Sakoda

**Affiliations:** 1 Laboratory of Microbiology, Department of Disease Control, Faculty of Veterinary Medicine, Hokkaido University, Kita 18, Nishi 9, Kita-ku, Sapporo 060-0818, Hokkaido, Japan; 2 One Health Research Center, Hokkaido University, Sapporo 060-0818, Hokkaido, Japan; 3 International Collaboration Unit, International Institute for Zoonosis Control, Hokkaido University, Sapporo 060-0818, Hokkaido, Japan; 4 Hokkaido University Institute for Vaccine Research and Development (HU-IVReD), Hokkaido University, Sapporo 060-0818, Hokkaido, Japan; 5 National Center for Veterinary Diagnostics No.1, Noi Bai Commune, Hanoi 12206, Vietnam; 6 Department of Animal Health, Kim Lien Ward, Hanoi 11520, Vietnam; 7 Kyoto Biken Laboratories, Inc., 16 Nijushi, Makishima-cho, Uji 611-0041, Kyoto, Japan

**Keywords:** AIV, candidate vaccine strain, chickens, clade 2.3.4.4b, egg products, H5 HPAIV, inactivated vaccine, laying hens, virus contamination

## Abstract

Background/Objectives: Biosecurity and stamping out are key control measures against H5 high pathogenicity avian influenza (HPAI) outbreaks. Vaccination in poultry is an additional tool to reduce disease risk and facilitate timely containment. This study aimed to establish a candidate vaccine strain against H5 HPAI in Asia and validate its protective efficacy. Methods: Based on genetic and antigenic analyses, a representative HPAI virus, A/duck/Vietnam/HU16-DD3/2023 (H5N1), collected in northern Vietnam, was selected to generate a candidate vaccine strain, rgPR8/VN23HA∆KRRK-NA (rgPR8/VN23; H5N1), using reverse genetics, followed by formulation of an inactivated oil-adjuvanted vaccine. Vaccine efficacy was evaluated by measuring humoral antibody responses after intramuscular vaccination and by assessing mortality and virus recovery following intranasal challenge with a clade 2.3.4.4b virus, A/Ezo red fox/Hokkaido/1/2022 (H5N1). Results were compared with those obtained using an antigenically homologous vaccine to the challenge strain and a Japanese stockpiled vaccine. Results: All vaccinated juvenile chickens developed sufficient immunity to survive the challenge at 21 days post-vaccination. The rgPR8/VN23 (H5N1) and homologous vaccines markedly reduced virus recovery, suggesting near-sterile protection, whereas low-titer viruses were transiently detected in chickens vaccinated with the stockpiled vaccine. The rgPR8/VN23 (H5N1) vaccine conferred clinical protection in juvenile chickens as early as 8 days post-vaccination. A single dose of the rgPR8/VN23 (H5N1) vaccine provided incomplete protection in laying hens, whereas a double-volume regimen improved protective efficacy. Conclusions: The rgPR8/VN23 (H5N1) vaccine conferred strong immunity to juvenile chickens; however, a refined vaccination strategy may be required to achieve complete protection in laying hens.

## 1. Introduction

High pathogenicity avian influenza (HPAI) continues to pose a global threat to the poultry industry, causing rapid mortality and substantial economic loss. According to the World Organisation for Animal Health (WOAH), from 2005 to 2024, HPAI resulted in the death of more than 663 million poultry worldwide, including infected and exposed birds, peaking at 146 million birds in 2022 [1]. Avian influenza viruses (AIVs) are classified into two pathotypes based on their pathogenicity in chickens, namely low pathogenicity AIV and high pathogenicity AIV (HPAIV), with the latter being the causative agent of HPAI [2]. AIVs possess two surface glycoproteins—hemagglutinin (HA) and neuraminidase (NA)—which are classified into 16 HA (H1–H16) and 9 NA (N1–N9) subtypes, respectively, based on their antiserum reactivity [2]. HPAIVs are limited to H5 or H7 subtypes [2]. Since the 2000s, H5 HPAIVs, particularly those derived from the A/goose/Guangdong/1/1996 (H5N1) lineage, have been prevalent for more than 20 years with substantial genetic diversity. Notably, H5 HPAI clade 2.3.4.4b strains have circulated mainly in Europe and Asia since 2016 [3] and have recently become a global concern [4].

Although strict biosecurity and stamping out are primary HPAI control measures, vaccination in poultry serves as a useful optional measure for reducing mortality, virus shedding, and virus spreading [5]. The WOAH also limits the use of vaccination to contexts where appropriate surveillance systems are implemented [2]. Given that HPAI control strategies vary widely across countries, including non-endemic and endemic countries, an ideal vaccine must be effective for both emergency deployment and routine protection. To support regional control of influenza outbreaks in animal health, the establishment of an influenza virus library through continuous surveillance has been proposed as an ideal preparedness measure for future control measures. One practical use of such a library is to provide virus strains for biologics development. For example, a Japanese stockpiled vaccine against H5 HPAIV for emergency use was prepared using this scheme. This vaccine comprises a non-pathogenic AIV, A/duck/Hokkaido/Vac-1/2004 (Dk/Hok/Vac-1/04; H5N1), and induces sufficient immunity in chickens to prevent mortality and clinical signs, although complete prevention of challenge-virus shedding was not achieved [6,7]. However, the antigenic drift in recently circulating H5 HPAIVs poses a significant risk of reduced protective efficacy for this traditional vaccine. Since 2020, clade 2.3.4.4b H5 HPAIVs have caused outbreaks across Europe and Asia [8,9,10]. H5N1 HPAIVs of this clade, isolated in Japan and the Republic of Korea during the winter of 2022–2023, were classified into subgroups G2b, G2c, and G2d within genotype group 2 [11]. Although the antigenic characteristics of these subgroups were similar to each other and to previous clade 2.3.4.4b viruses, they were antigenically distinct from the stockpiled vaccine strain Dk/Hok/Vac-1/04 (H5N1) [12]. This antigenic mismatch poses a challenge to maintaining vaccine effectiveness.

A vaccine derived from an endemic strain should provide high protective efficacy against currently circulating field strains. Therefore, updating vaccine strains to ensure antigenic matching with circulating strains is essential for preparedness against HPAI outbreaks in Asia. This study aimed to establish a candidate vaccine strain through several steps, including enrichment of the AIV library by field surveillance, analysis of the genetic and antigenic characteristics of HPAIVs, and evaluation of vaccine protective efficacy. Protective efficacy was assessed using a challenge study with a clade 2.3.4.4b HPAIV, A/Ezo red fox/Hokkaido/1/2022 (Fox/Hok/1/22; H5N1) [13]. Additionally, an inactivated vaccine derived from a recombinant strain carrying the HA and NA genes of Fox/Hok/1/22 (H5N1) with a modified HA cleavage site, designated NIID-002 (A/Ezo red fox/Hokkaido/1/2022) (NIID-002; H5N1) [14], and an inactivated vaccine derived from Dk/Hok/Vac-1/04 (H5N1) virus [6] were included for comparative evaluation of protective efficacy. Given the need to evaluate the candidate vaccine for both routine protection and emergency deployment, three independent animal experiments were conducted. First, to simulate routine vaccination programs in HPAI-endemic countries, protective efficacy was assessed in juvenile chickens, the primary target population for routine vaccination. Second, to evaluate suitability for emergency deployment, particularly for rapid containment during widespread outbreaks, the earliest onset of protective immunity was determined in juvenile chickens. Third, given that emergency vaccination should apply to all poultry populations, including juvenile and adult birds, the study was extended to evaluate vaccine efficacy in approximately 40-week-old laying hens. This assessment included initial single-dose testing followed by optimization using a double-volume simultaneous regimen. Overall, these findings contribute to a deeper understanding of the efficacy and limitations of the oil-adjuvanted vaccine in both juvenile and adult birds and support improved HPAI control strategies using inactivated vaccines.

## 2. Materials and Methods

### 2.1. Sample Collection, Virus Isolation, and Sequencing

To collect HPAIVs circulating in the field, AIV surveillance was conducted in northern Vietnam in 2019, 2021, 2023, and 2024, and in southern Vietnam in 2019 and 2021. Oropharyngeal and cloacal swabs from live poultry, including chickens, ducks, and Muscovy ducks, and environmental swabs from floors or water containers were collected at live bird markets, farms, and poultry transport stations. Samples were preserved in viral transport medium prepared in-house at the National Center of Veterinary Diagnostics, Hanoi, Vietnam, using minimum essential medium (MEM; Gibco, Thermo Fisher Scientific, Waltham, MA, USA), penicillin–streptomycin (10,000 IU/mL; Gibco), HEPES buffer (Sartorius, Göttingen, Germany), and 35% bovine serum albumin (Sigma-Aldrich, St. Louis, MO, USA).

To detect AIV genes, 10 individual swab samples were pooled and screened via reverse transcription quantitative polymerase chain reaction (RT-qPCR) targeting the M gene, following WOAH guidelines [2], at the National Center of Veterinary Diagnostics, Hanoi, Vietnam. Samples positive on RT-qPCR were inoculated into 10-day-old embryonated chicken eggs for virus isolation in the Biosafety Level 3 (BSL-3) facility at the Laboratory of Microbiology, Faculty of Veterinary Medicine, Hokkaido University, Hokkaido, Japan.

From the isolated H5 HPAIVs, representative H5 HPAIVs were selected for whole-genome sequencing using next-generation sequencing based on geographic region and isolation year. For Oxford Nanopore sequencing, libraries were generated using the NEB Ultra II End Repair/dA-Tailing Module (New England Biolabs, Ipswich, MA, USA). The prepared libraries were subsequently sequenced on a Flongle flow cell using either the Direct cDNA Sequencing Kit (Oxford Nanopore Technologies, Oxford, UK) or the Ligation Sequencing Kit V14 (Oxford Nanopore Technologies). Raw reads were processed, mapped, and assembled using FluGAS v2 (World Fusion, Tokyo, Japan). The sequence profiles of the H5 HPAIVs were registered in the Global Initiative on Sharing All Influenza Data (GISAID).

### 2.2. Phylogenetic Analysis

The nucleotide sequences of the representative isolates, along with reference H5 HPAIV sequences obtained from the GISAID database, were analyzed phylogenetically by MEGA 7 (version 7.0.26) [15]. Phylogenetic trees were constructed using the maximum likelihood method with the Tamura–Nei nucleotide substitution model. Rate heterogeneity among sites was modeled using a gamma distribution with four discrete categories, and branch support was evaluated using 1000 bootstrap replicates.

### 2.3. Hemagglutination Inhibition (HI) Test

Sera were heat-inactivated at 56 °C for 30 min and subsequently adsorbed with chicken red blood cells (cRBCs) following the Japanese standards for veterinary biological products. Sera were mixed with 10% cRBCs at a 1:3 ratio (1 volume serum and 3 volumes of 10% cRBCs), incubated at 4 °C overnight, and centrifuged (1000× *g*, 5 min). The supernatant was collected as a fourfold diluted serum. The HI test was performed according to the WOAH guidelines, and HI titers were defined as the highest serum dilution that completely inhibited four hemagglutination (HA) units of antigen [2].

### 2.4. Antigenic Analysis

The antigenic characteristics of the representative viruses were evaluated by a cross-HI test with chicken hyperimmune antisera, as previously described [16]. The antigen panel included the following antigens: A/duck/Vietnam/HU16-DD3/2023 (Dk/VN/DD3/23; H5N1), A/duck/Vietnam/HU16-NS82/2023 (Dk/VN/NS82/23; H5N1), A/Muscovy duck/Vietnam/HU14-GV50/2021 (Mdk/VN/GV50/21; H5N8), A/chicken/Vietnam/HU11-903/2019 (Ck/VN/903/19; H5N6), A/duck/Vietnam/HU12-971/2019 (Dk/VN/971/19; H5N6), and A/chicken/Vietnam/HU12-657/2019 (Ck/VN/657/19; H5N1), which were isolated in this study. Reference antigens from other H5 clades were also analyzed. Viruses from clade 2.3.4.4b included A/Eurasian wigeon/Hokkaido/Q71/2022 (EW/Hok/Q71/22; H5N1), A/white-tailed eagle/Hokkaido/22-RU-WTE-2/2022 (WTE/Hok/R22/22; H5N1), Fox/Hok/1/22 (H5N1), and A/northern pintail/Hokkaido/M13/2020 (Np/Hok/M13/20; H5N1). Clade 2.3.4.4c was represented by A/chicken/Kumamoto/1-7/2014 (Ck/Kum/1-7/14; H5N8). Clade 2.3.4.4e was represented by A/black swan/Akita/1/2016 (Bs/Aki/1/16; H5N6). Clade 2.3.4.4 was represented by A/chicken/Vietnam/HU4-42/2015 (Ck/VN/42/15; H5N6). Clade 2.3.4.4g was represented by A/Muscovy duck/Vietnam/HU7-20/2017 (Mdk/VN/20/17; H5N6). Clade 2.3.4 was represented by A/peregrine falcon/Hong Kong/810/2009 (Pfal/HK/810/09; H5N1). Clade 2.3.2.1e, formerly 2.3.2.1c [17], was represented by A/duck/Vietnam/HU3-836/2015 (Dk/VN/386/15; H5N1). Clade 1.1 was represented by A/Muscovy duck/Vietnam/OIE-559/2011 (Mdk/VN/559/11; H5N1). The stockpiled strain Dk/Hok/Vac-1/04 (H5N1) was included as a reference. The antisera panel included EW/Hok/Q71/22, WTE/Hok/R22/22, Bs/Akita/1/16, Ck/Kum/1-7/14, Dk/VN/20/17, Dk/VN/386/15, and Pfal/HK/810/09. Hyperimmune serum against the Dk/VN/DD3/23 (H5N1) strain was prepared according to Kida and Yanagawa [16]. Based on the cross-HI test results, antigenic cartography was generated using the Racmac application in the R program (version 4.4.2) [18]. HI titers were transformed to a log_2_ scale to calculate antigenic distances, where each twofold change in titer corresponded to one unit of antigenic distance. Antigens and antisera were positioned in two-dimensional space using multidimensional scaling.

### 2.5. Animals, Cells, and Viruses

White Leghorn chickens were home-bred from embryonated eggs and raised to 7 weeks old at the Experimental Animal Facility, Faculty of Veterinary Medicine, Hokkaido University. Chickens were then transferred to self-contained isolator units (Tokiwa Kagaku Kikai, Tokyo, Japan) at the BSL-3 facility, Faculty of Veterinary Medicine, Hokkaido University. Additionally, 40-week-old White Leghorn laying hens were obtained from Hokuryo, Hokkaido, Japan. The hens were individually housed in the same type of self-contained isolator units within the BSL-3 facility. Environmental enrichment was achieved by installing stainless steel mirrors in each isolator to reduce stress. All chickens used in this study had no prior vaccination history and lacked maternally derived antibodies, because vaccination against AIVs is not implemented in Japan.

Madin–Darby canine kidney (MDCK) cells were cultured at 37 °C in MEM (Shimadzu Diagnostics Corporation, Tokyo, Japan) supplemented with 0.3 mg/mL L-glutamine (Nacalai Tesque, Kyoto, Japan), 100 U/mL penicillin G, 0.1 mg/mL streptomycin (both from Meiji Seika Pharma, Tokyo, Japan), 8 µg/mL gentamicin (Takata Pharmaceutical, Saitama, Japan), and 10% fetal bovine serum (Merck KGaA, Darmstadt, Germany). Human embryonic kidney 293T (HEK293T) cells were maintained at 37 °C in a humidified atmosphere containing 5% CO_2_ in pyruvate-free Dulbecco’s modified Eagle’s medium (Gibco), supplemented with the same antibiotics and 10% fetal bovine serum (Nichirei Biosciences, Tokyo, Japan).

The Fox/Hok/1/22 (H5N1) virus, previously isolated from a deceased Ezo red fox (*Vulpes vulpes schrencki*) [13], served as the challenge strain. For comparison, the NIID-002 (H5N1) virus [14], provided by the National Institute of Infectious Diseases, Japan, served as the vaccine strain. The Dk/VN/DD3/23 (H5N1) virus, isolated from an apparently healthy duck at a live bird market in northern Vietnam, served as the parental strain for establishing the candidate vaccine strain. All three viruses were propagated in 10-day-old embryonated chicken eggs. Viral presence in allantoic fluid was confirmed using the HA test, and the 50% egg infectious dose (EID_50_) was determined for each strain. Additionally, the 50% chicken lethal dose (CLD_50_) was determined for the challenge strain Fox/Hok/1/22 (H5N1).

### 2.6. Generation and Evaluation of Vaccine Strain

Reverse genetics was used to generate a recombinant virus incorporating H5 HA and N1 NA from the representative field strain with the internal genes derived from A/Puerto Rico/8/1934 (PR8; H1N1). To reduce virulence, the HA gene was cloned into the pGEM-T Easy vector (Promega, Madison, WI, USA) and mutated at the HA cleavage site. Polybasic amino acids (EKRRKR|GLF) were mutated to the monobasic amino acid threonine (ETR|GLF) using the KOD-Plus Mutagenesis Kit (TOYOBO, Osaka, Japan). The modified HA gene was subcloned, and the NA gene was directly cloned into the pHW2000 vector [19]. Plasmids containing the PR8-derived internal genes were utilized. The eight pHW2000 plasmids were transfected into the co-cultured MDCK and HEK293T cells. At 72 h post-transfection, the supernatant was collected and inoculated into 10-day-old embryonated chicken eggs for virus propagation.

To assess growth kinetics, the vaccine strains were inoculated into 10-day-old embryonated chicken eggs at doses of 2.0 and 4.0 log_10_ EID_50_/0.1 mL per egg. Eggs were collected at 12, 24, 36, 48, 60, and 72 h post-inoculation to measure viral titers (expressed as EID_50_).

To confirm the low pathogenicity of the vaccine strains, the intravenous pathogenicity index (IVPI) was determined following the WOAH guidelines [2]. Briefly, 6-week-old White Leghorn chickens (*n* = 8 per vaccine strain) were intravenously inoculated via the wing vein with 0.1 mL of a 1:10 dilution of infective allantoic fluid. The chickens were observed daily for 10 days, and clinical signs were scored as follows: 0 for no sign (normal); 1 for a single sign (respiratory symptom, depression, diarrhea, cyanosis, edema, or nervous symptom; sick); 2 for multiple signs (seriously sick); and 3 for death. Birds reaching humane endpoints, such as severe clinical signs or inability to eat or drink, were euthanized to prevent distress; death was confirmed before disposal. The IVPI was calculated as the mean score per chicken, with an index score > 1.2 used as the threshold for classifying viruses as HPAIV.

To evaluate genetic stability, recombinant vaccine strains were serially passaged five times in 10-day-old embryonated chicken eggs. HA-positive allantoic fluid from each round was used to inoculate the subsequent passage. Viral RNA was extracted from harvested allantoic fluid and subjected to next-generation sequencing to assess potential nucleotide mutations across the viral genome. Growth stability was further evaluated by determining viral titers in embryonated chicken eggs, expressed as EID_50_. The geometric means of the EID_50_ before and after serial passages were compared to assess growth stability.

### 2.7. Vaccine Preparation

Whole harvested allantoic fluid containing each vaccine strain was inactivated by incubation with formalin at a final concentration of 0.2% for 3 days at 4 °C. Three successive serial passages in 10-day-old embryonated chicken eggs were performed to confirm virus inactivation. Trial vaccines were produced by mixing with an oil adjuvant, as previously described [6]. Based on HA titers, the inactivated virus suspension was diluted with sterile phosphate-buffered saline (PBS) to obtain a final concentration of 633.5 HA units/0.5 mL. The suspensions were then mixed with an oil adjuvant. Using an ultra-homomixer (PRIMIX, Osaka, Japan), the emulsion was homogenized to produce a water-in-oil vaccine.

### 2.8. Assessment of Vaccine Protective Capacity in Juvenile Chickens

Forty 4-week-old White Leghorn chickens were randomly divided into 4 groups of 10 chickens each, including three vaccinated groups and one unvaccinated group. Chickens were intramuscularly vaccinated with the vaccine developed in this study, the NIID-002 (H5N1) vaccine, or the Dk/Hok/Vac-1/04 (H5N1) vaccine at a single dose of 633.5 HA units/0.5 mL. Blood was collected weekly to monitor the antibody responses. At 21 days post-vaccination (dpv) (7-week-old), all chickens were intranasally challenged with 100 CLD_50_ of Fox/Hok/1/22 (H5N1), equivalent to 6.2 log_10_ EID_50_/0.1 mL. Clinical signs and mortality were monitored daily until 14 days post-challenge (dpc). Birds reaching humane endpoints, such as severe clinical signs or inability to eat or drink, were euthanized to prevent distress; death was confirmed before disposal. Oropharyngeal and cloacal swab samples were collected at 2, 3, 5, and 7 dpc to assess viral shedding.

### 2.9. RT-qPCR

A subset of oropharyngeal and cloacal swab samples was obtained from chickens vaccinated with the vaccine developed in this study, the Dk/Hok/Vac-1/04 (H5N1) vaccine, and unvaccinated chickens (5 chickens per group). Viral RNA was extracted using the MagMAX-96 Total RNA Isolation Kit (Thermo Fisher Scientific, Waltham, MA, USA), according to the manufacturer’s instructions. RT-qPCR was performed using the Thunderbird Probe One-Step qRT-PCR Kit (TOYOBO), and each sample was analyzed in duplicate according to the manufacturer’s instructions. The RT-qPCR assay targeted the M gene using the primer and probe sequences shown in Table S1.

### 2.10. Determination of the Earliest Onset of Protective Immunity in Juvenile Chickens

Twenty 35-day-old White Leghorn chickens were randomly divided into 5 groups of 4 chickens each. Four groups were intramuscularly vaccinated with a single dose of the vaccine developed in this study at 35, 39, 41, and 43 days of age, corresponding to 14, 10, 8, and 6 days before the challenge. The remaining chickens constituted the unvaccinated group. All chickens were bled at 49 days of age (7-week-old) to evaluate antibody responses immediately before the challenge and were subsequently intranasally inoculated with 100 CLD_50_/0.1 mL of Fox/Hok/1/22 (H5N1). Clinical signs and mortality were monitored daily for 14 days. Birds reaching humane endpoints, such as severe clinical signs or inability to eat or drink, were euthanized to prevent distress; death was confirmed before disposal. Oropharyngeal and cloacal swab samples were collected at 2, 3, 5, and 7 dpc to assess viral shedding.

### 2.11. Evaluation of Vaccine Efficacy in the Laying Hens

Two independent experimental rounds were conducted to evaluate vaccination regimens in 40-week-old White Leghorn hens. Both rounds included unvaccinated and vaccinated groups, with 4 hens per group. In round 1 (*n* = 8), the efficacy of a single vaccine dose (633.5 HA units/0.5 mL) was assessed. In round 2 (*n* = 12), simultaneous vaccinations with a single- and double-volume dose (2 × 0.5 mL total) were compared. The hens were intramuscularly vaccinated, and blood samples were collected weekly to monitor antibody responses. All hens were intranasally challenged with 100 CLD_50_/0.1 mL of Fox/Hok/1/22 (H5N1) at 21 dpv. Clinical signs and mortality were monitored daily. Birds reaching humane endpoints, such as severe clinical signs or inability to eat or drink, were euthanized to prevent distress; death was confirmed before disposal. Oropharyngeal and cloacal swab samples were collected at 2, 3, 5, and 7 dpc in round 1 and at 2, 3, 4, 5, and 7 dpc in round 2. Eggs laid by hens were also collected daily from 1 to 7 dpc to detect H5 HPAIV contamination. Eggshells were wiped with cotton swabs soaked in viral transport medium. Egg yolks and egg whites were collected separately.

### 2.12. Virus Titration

Oropharyngeal and cloacal swab samples were serially diluted 10-fold in MEM. Confluent MDCK cell monolayers were incubated with the diluted swab samples for 1 h at 35 °C. The inoculum was discarded, and cells were washed with sterile PBS. The cultures were then maintained in serum-free MEM supplemented with 1.0 µg/mL acetylated trypsin (Merck KGaA) at 35 °C for 72 h. Cytopathic effects were observed and recorded to calculate the 50% tissue culture infectious dose (TCID_50_).

Eggshell swab and egg white samples were serially diluted 10-fold in sterile PBS. Egg yolk was prediluted 1:2 in sterile PBS before performing serial 10-fold dilutions because of its high viscosity. The diluted egg-derived samples were then inoculated into 10-day-old embryonated chicken eggs. After incubation at 35 °C for 48 h, allantoic fluid was harvested, and the HA test was performed to calculate EID_50_ values. All viral titers were calculated using the Reed and Muench method [20].

### 2.13. Statistical Analysis

GraphPad Prism version 10.1.2 (GraphPad Software, San Diego, CA, USA) was used for statistical analysis. Viral growth stability was analyzed using the Wilcoxon signed-rank test. Antibody titers across groups were compared using one-way analysis of variance (ANOVA), followed by Tukey’s honestly significant difference post hoc test for pairwise comparisons. Data obtained from two groups were analyzed using Student’s *t*-test. Survival rates were analyzed using the log-rank test. A *p*-value of <0.05 was considered statistically significant.

## 3. Results

### 3.1. AIV Surveillance in Vietnam from 2019 to 2024

Surveillance in poultry was conducted in northern Vietnam in 2019, 2021, 2023, and 2024, and in southern Vietnam in 2019 and 2021 (Table S2). A total of 749 AIVs were isolated from 6034 collected samples, of which 108 were H5 HPAIVs, including 37 H5N6 viruses in 2019 and 2021; 21 H5N8 viruses in 2021; and 50 H5N1 viruses in 2019, 2023, and 2024. Other isolated subtypes included H9N2, H6N6, H3N2, H3N8, H3N4, H6N2, H6N3, H6N4, H9N4, and H10N3.

### 3.2. Genetic Analysis of H5 HA Genes of HPAIVs

**Figure 1:**
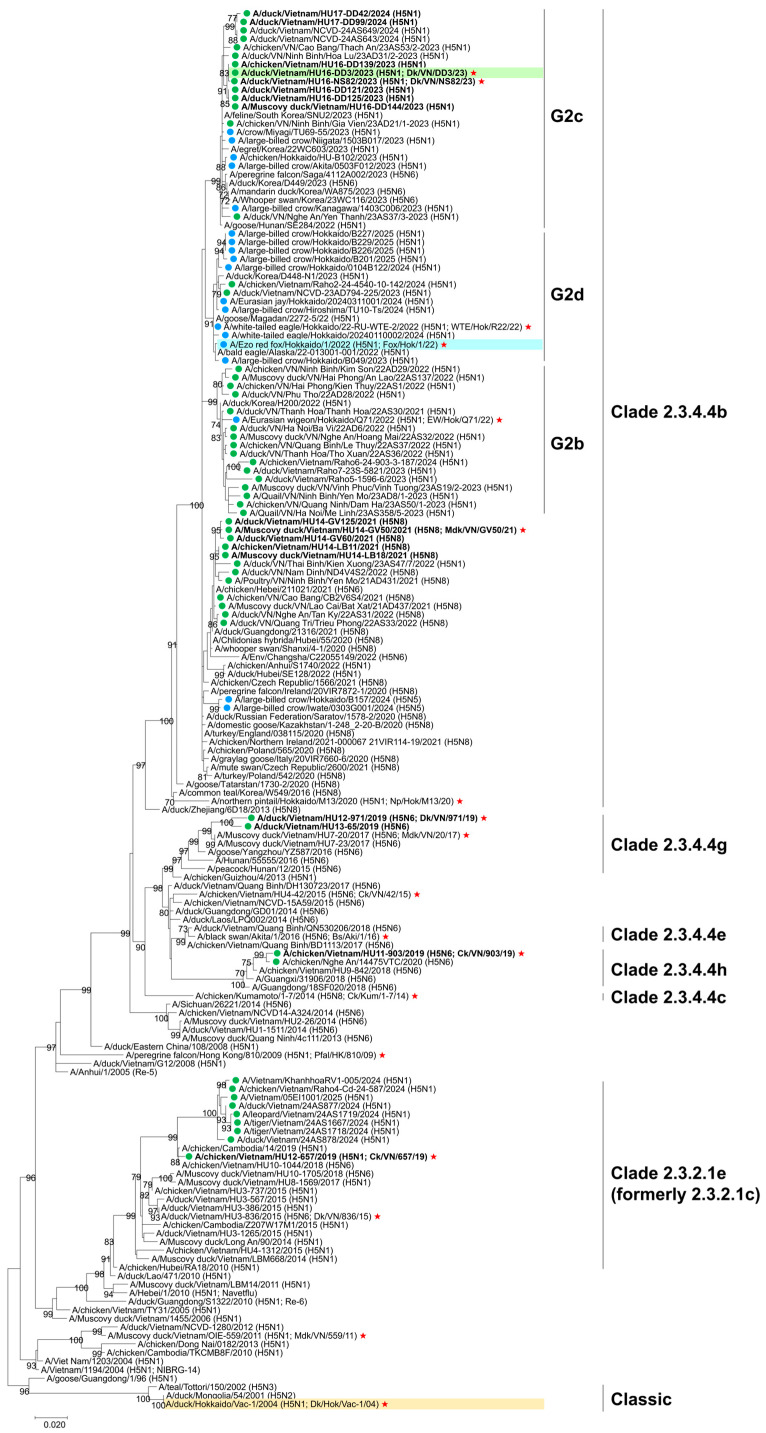
Phylogenetic tree of hemagglutinin genes of H5 avian influenza viruses analyzed using the maximum likelihood method with MEGA 7. The scale bar indicates nucleotide substitutions per site, and node values show bootstrap support (>50%, 1000 replicates). Vietnamese isolates are marked with green circles (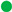), and isolates from this study are shown in bold. Japanese isolates from the 2022–2023, 2023–2024, and 2024–2025 seasons are marked with blue circles (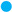). Strains highlighted in green, blue, and orange represent the selected strains of the new vaccine candidate, challenge, and stock vaccine strains, respectively. Isolates selected for antigenic analysis are marked with red stars (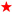).

HA genes from 17 H5 HPAIVs obtained through surveillance were sequenced for phylogenetic analysis (Table S3). The analysis showed that isolates in 2019 belonged to clades 2.3.2.1e (formerly 2.3.2.1c), 2.3.4.4h, and 2.3.4.4g (Figure 1). Clade 2.3.4.4h and 2.3.4.4g viruses were genetically related to strains previously isolated in Vietnam and China but have not been detected since 2021. Clade 2.3.2.1e viruses, genetically close to strains isolated in Vietnam and Cambodia, continued to circulate with slight genetic variability. Isolates from Vietnam in 2023–2024 belonged to clade 2.3.4.4b and subgroup G2c and were genetically similar to those from Japan and the Republic of Korea. Collectively, these results indicate significant genetic diversity among H5 HPAIVs circulating in the field. All analyzed H5 HPAIVs were genetically distinct from classic H5 strains, including the Japanese stockpiled vaccine strain Dk/Hok/Vac-1/04 (H5N1).

### 3.3. Antigenic Analysis of H5 HPAIVs

**Figure 2:**
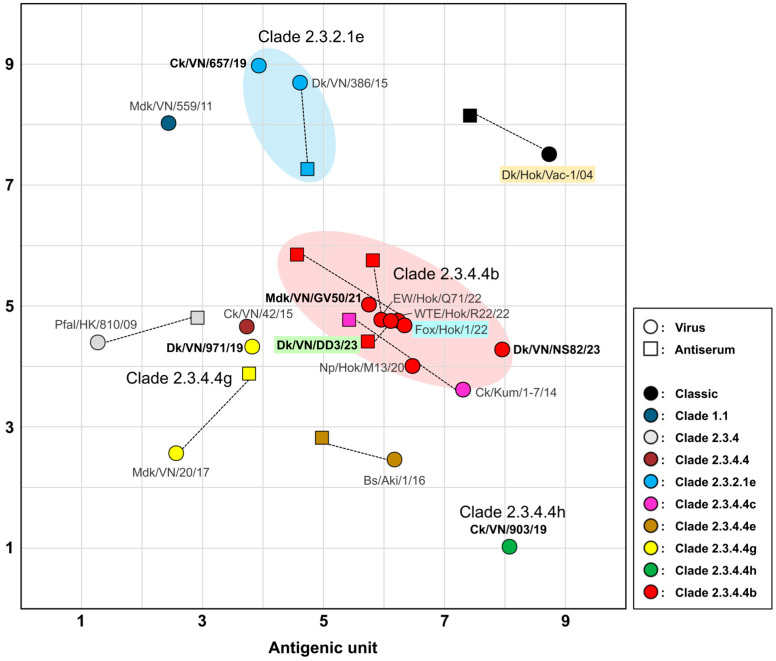
Antigenic cartography of H5 avian influenza viruses. The vertical and horizontal axes represent antigenic distances. Grid lines correspond to 1 unit, equivalent to a twofold difference in hemagglutination inhibition titer. Circles indicate viruses, and squares indicate sera. Colors indicate different clades. Viruses isolated in this study are shown in bold. Strains highlighted in green, blue, and orange correspond to the selected strains of the new vaccine candidate, challenge, and stock vaccine strains, respectively.

The antigenicity of H5 HPAIVs isolated from 2019 to 2024 was analyzed (Table S4) and visualized using antigenic cartography (Figure 2). On the cartograph map, clade 2.3.4.4b viruses displayed antigenic similarity, with distances of <1 antigenic unit. These viruses also showed antigenic proximity to strains belonging to clade 2.3.4.4c, Ck/Kum/1-7/14 (H5N8), and the precursor clade 2.3.4.4, Ck/VN/42/15 (H5N8). The Dk/VN/971/19 (H5N6) strain, belonging to clade 2.3.4.4g, showed antigenic closeness to viruses of clades 2.3.4.4b and 2.3.4.4 despite a greater than fourfold difference in HI titer compared with the Mdk/VN/20/17 (H5N8) isolate of clade 2.3.4.4g. Clade 2.3.2.1e viruses formed an antigenically distinct cluster separate from clade 2.3.4.4b viruses, as shown on the antigenic map. Similarly, the clade 2.3.4.4h virus Ck/VN/903/19 (H5N6) was antigenically distinct from the other analyzed isolates. Notably, all analyzed isolates were antigenically divergent from the Dk/Hok/Vac-1/04 (H5N1) strain. Collectively, these findings highlight the antigenic distinctions among the circulating H5 HPAIVs in the field.

### 3.4. Generation and Characterization of Vaccine Strain

The Dk/VN/DD3/23 (H5N1) virus was selected for vaccine development based on genetic and antigenic analyses. A recombinant strain, rgPR8/VN23HA∆KRRK-NA (rgPR8/VN23; H5N1), was rescued using reverse genetics. Successful virus rescue was confirmed by efficient propagation in embryonated chicken eggs with positive HA activity. Sequence analysis confirmed that the virus lacked polybasic amino acids at the HA cleavage site and had no unexpected mutations. Cross-HI titers of rgPR8/VN23 (H5N1) and Dk/VN/DD3/23 (H5N1) strains against antisera of Dk/VN/DD3/23 (H5N1) were both 640 HI, indicating that the rgPR8/VN23 (H5N1) strain was not antigenically different from Dk/VN/DD3/23 (H5N1), thus confirming antigenic integrity.

Chickens intravenously inoculated with the rgPR8/VN23 (H5N1) or NIID-002 (H5N1) vaccine strain remained clinically normal during 10 days of observation. Both vaccine strains had an IVPI of 0.0 (Table S5). These results confirm the successful attenuation and safety of both the rgPR8/VN23 (H5N1) and NIID-002 (H5N1) vaccine strains.

The rgPR8/VN23 (H5N1) vaccine strain exhibited high growth properties in embryonated chicken eggs at inoculation doses of both 2.0 and 4.0 log_10_ EID_50_/0.1 mL. Viral titers increased rapidly within the first 24 h of inoculation and remained within 9.3–10.3 log_10_ EID_50_/mL from 24 to 72 h post-inoculation (Figure S1). Although the NIID-002 (H5N1) vaccine strain, which carries the HA and NA genes of Fox/Hox/22 (H5N1) and six internal genes of PR8 (H1N1), showed lower growth during the early stages of infection, its growth kinetics were comparable to those of the rgPR8/VN23 (H5N1) strain after 48 h. These data indicate that the rgPR8/VN23 (H5N1) and NIID-002 (H5N1) vaccine strains have high growth potential in eggs and meet key requirements for vaccine strains: low pathogenicity in chickens and high growth potential in embryonated chicken eggs.

To ensure suitability for mass production, the recombinant vaccine strains were monitored for genetic and growth stability. After five serial passages in embryonated chicken eggs, no nucleotide mutations were detected across all eight gene segments of either the rgPR8/VN23 (H5N1) or NIID-002 (H5N1) vaccine strains (Table S6). Viral titers at passage 5 remained within a similar range to those at passage 1 for both strains (Figure S2). The geometric mean titers (GMTs) for the rgPR8/VN23 (H5N1) strain were 9.7 and 9.5 log_10_ EID_50_ at passages 1 and 5, respectively (*p* > 0.05), whereas those for the NIID-002 (H5N1) strain were 8.9 and 9.6 log_10_ EID_50_ at passages 1 and 5, respectively (*p* > 0.05). No consistent decline was observed, indicating biologically comparable growth characteristics. These findings support the maintenance of genetic integrity and growth characteristics during five serial propagations.

### 3.5. Vaccine Protective Capacity in Juvenile Chickens

**Figure 3:**
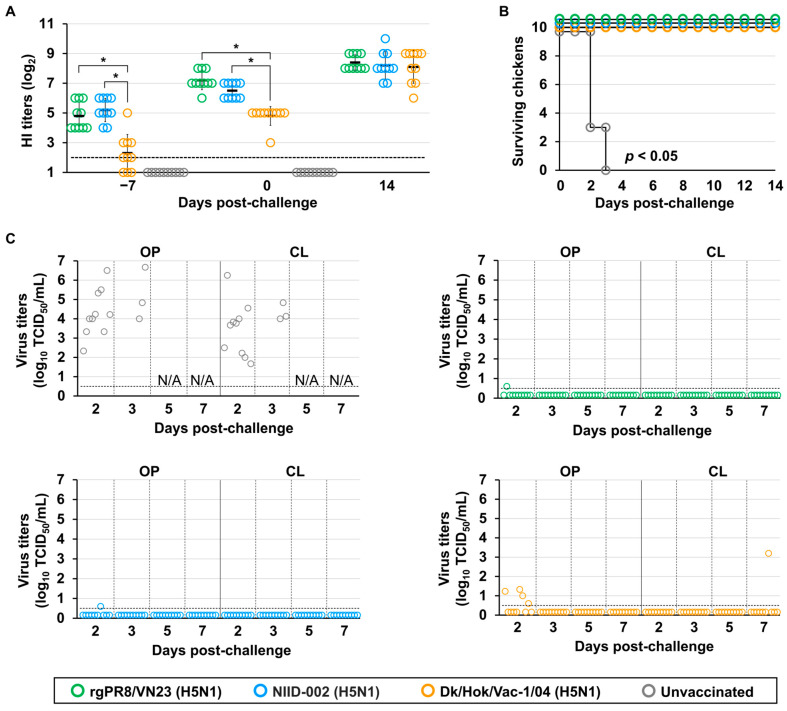
Vaccine efficacy against H5 high pathogenicity avian influenza in juvenile chickens: (**A**) Immunological responses against the challenge strain, A/Ezo red fox/Hokkaido/1/2022 (H5N1). The *X*-axis displays time in days; −7 indicates the time point corresponding to 14 days post-vaccination (dpv); 0 corresponds to 21 dpv; and 14 indicates days post-challenge. Open circles represent individual hemagglutination inhibition (HI) titers. Bars represent geometric mean titers. The horizontal dashed line indicates the detection limit (2 log_2_). The asterisk indicates a statistically significant difference (*p* < 0.05). (**B**) Survival rates of chickens after challenge. Survival curves were analyzed using the Kaplan–Meier method, and differences between vaccinated and unvaccinated groups were determined using the log-rank test (*p* < 0.05). (**C**) Virus recovery from oropharyngeal (OP) and cloacal (CL) swabs after challenge. Virus titers are expressed as log_10_ of the 50% tissue culture infectious dose (TCID_50_/mL). The horizontal dashed line indicates the detection limit (0.5 log_10_ TCID_50_/mL). N/A: not available due to chicken mortality.

After preparing the inactivated oil-adjuvanted vaccines, immunogenicity was assessed by monitoring antibody titers (Figure S3). The three vaccines derived from the rgPR8/VN23 (H5N1), NIID-002 (H5N1), and Dk/Vac-1/04 (H5N1) strains elicited immune responses against their homologous strains (Figure S4) and the challenge strain (Figure 3A). HI antibody titers before vaccination and at 7 dpv were <2 log_2_. At 14 dpv, chickens vaccinated with rgPR8/VN23 (H5N1) or NIID-002 (H5N1) vaccines showed comparable antibody levels against the challenge strain, with GMTs of 4.8 ± 0.92 log_2_ and 5.2 ± 0.79 log_2_, respectively. Compared with the other vaccine groups, chickens vaccinated with the Dk/Hok/Vac-1/04 (H5N1) vaccine showed significantly lower antibody titers against the challenge strain (*p* < 0.05). Only one of 10 chickens reached 5 log_2_, whereas the others showed titers of 2–3 log_2_ or undetectable levels. At 21 dpv, the GMT of HI antibodies in the rgPR8/VN23 (H5N1) vaccine group was 7.2 ± 0.63 log_2_, which was higher than that of the NIID-002 (H5N1) vaccine group (6.5 ± 0.53 log_2_). However, this difference was not statistically significant (*p* > 0.05). The GMT of HI antibody in chickens vaccinated with the Dk/Hok/Vac-1/04 (H5N1) vaccine was 4.8 ± 0.63 log_2_, which was significantly lower than that of the other vaccine groups. Collectively, these data indicate that the HI titers increased by 14 dpv. However, the antibody titers against the challenge strain were lower in the Dk/Hok/Vac-1/04 (H5N1) vaccine group, potentially reflecting antigenic differences between the clade 2.3.4.4b and classic H5N1 viruses. At 14 dpc, no significant differences in GMTs of HI antibodies were observed among the three vaccinated groups (*p* > 0.05), and within each group, HI titers against the homologous and challenge strains were comparable.

Vaccine efficacy was evaluated based on mortality following challenge with the Fox/Hok/1/22 (H5N1) virus. All chickens in the unvaccinated group died within 3 days, whereas all vaccinated chickens survived until 14 dpc (Figure 3B), demonstrating that all three vaccines conferred strong preventive immunity against clinical symptoms after HPAIV infection.

Virus shedding from both oropharyngeal and cloacal swabs was observed in all unvaccinated chickens. Viral loads varied among individuals, ranging from 2.3 to 6.7 log_10_ TCID_50_/mL in oropharyngeal samples and from 1.7 to 6.3 log_10_ TCID_50_/mL in cloacal samples (Figure 3C). Conversely, detectable virus (>0.5 log_10_ TCID_50_/mL) was not confirmed in most chickens vaccinated with the rgPR8/VN23 (H5N1) and NIID-002 (H5N1) vaccines in either the oropharynx or cloaca during sampling. Transient virus shedding was detected in oropharyngeal samples from one chicken in each of the rgPR8/VN23 (H5N1) and NIID-002 (H5N1) vaccine groups at 2 dpc, with a very low virus titer (0.6 log_10_ TCID_50_/mL). Infectious virus was not detected in cloacal swabs from these groups. In the Dk/Hok/Vac-1/04 (H5N1) vaccine group, infectious viruses were recovered from 4 of the 10 chickens in oropharyngeal swabs at 2 dpc, with titers of 0.6–1.3 log_10_ TCID_50_/mL. Of these 4 chickens, three showed no further virus shedding, whereas virus shedding reappeared in the cloacal swab of one chicken at 7 dpc, with a titer of 3.2 log_10_ TCID_50_/mL. Overall, all three vaccines reduced viral titers in swab samples compared with the unvaccinated group. The rgPR8/VN23 (H5N1) and NIID-002 (H5N1) vaccines demonstrated superior efficacy by markedly reducing virus shedding, resulting in near-sterile protection, whereas the Dk/Hok/Vac-1/04 (H5N1) vaccine provided only partial reduction.

To investigate the persistence of viral genes, the M gene was detected using RT-qPCR in a subset of swab samples. Given the comparable protection conferred by the rgPR8/VN23 (H5N1) and NIID-002 (H5N1) vaccines, subsequent analyses compared the rgPR8/VN23 (H5N1) vaccine with the Dk/Hok/Vac-1/04 (H5N1) vaccine. Swab samples collected before the challenge served as negative controls for M detection via RT-qPCR and showed Ct values ≥ 37. Samples from the unvaccinated group (positive controls) showed strong signals with Ct values < 35, consistent with infectious virus titers (Figure S5). In the rgPR8/VN23 (H5N1) vaccine group, M was detected with Ct values of 35–37, although infectious virus was not recovered. In the Dk/Hok/Vac-1/04 (H5N1) vaccine group, low-level M detection (Ct value 35–37) was also observed, in addition to strong signals (Ct values < 35) in samples positive for infectious virus.

### 3.6. Vaccine Protection Onset in Juvenile Chickens

**Figure 4:**
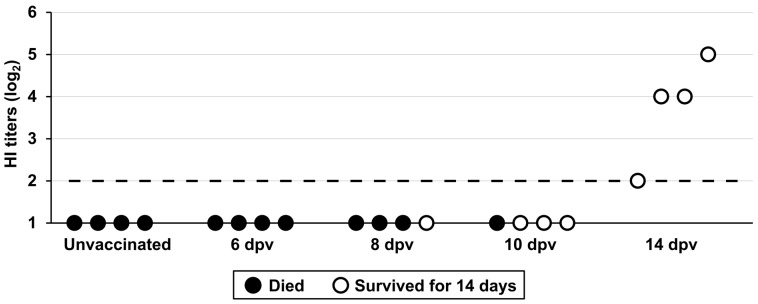
Immune responses and protection status at various days post-vaccination (dpv). The *X*-axis displays different groups, including unvaccinated and vaccinated chickens challenged at 6, 8, 10, and 14 dpv. Individual hemagglutination inhibition (HI) titers (log_2_) were measured at challenge. Each dot represents a single chicken. White and black dots denote survival and mortality, respectively. The horizontal dashed line indicates the detection limit (2 log_2_).

The onset of vaccine protection was determined as the earliest time after vaccination at which chickens were protected against mortality following the H5 HPAIV challenge. All vaccinated chickens challenged at 6 dpv died within 3 days (Figure S6A), similar to the unvaccinated group. When challenged at 8 dpv, one of 4 chickens survived, indicating partial immunity at this time point. Protection rates increased substantially in the group challenged at 10 dpv, with 3 of 4 chickens surviving. Optimal protection was observed at 14 dpv, when all chickens survived. HI titers were below the detection limit (2 log_2_) in serum samples collected at 6, 8, and 10 dpv, whereas antibodies were detectable in serum samples collected at 14 dpv, ranging from 2 to 5 log_2_ (Figure 4). Infectious viruses were not detected in the 14 dpv group, contrasting with virus recovery in chickens challenged at 6, 8, and 10 dpv (Figure S6B).

### 3.7. Vaccine Efficacy in Laying Hens

**Figure 5:**
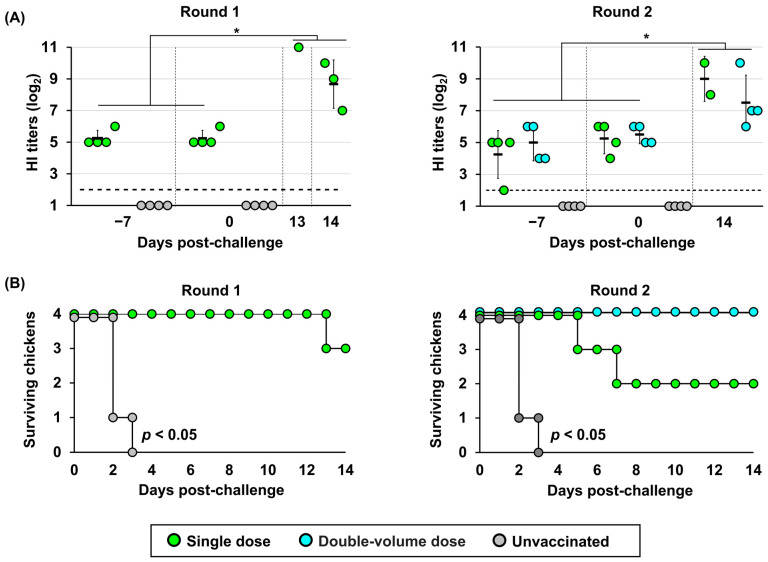
Immunogenicity and survival rates of hens vaccinated using different regimens: (**A**) Immunological responses against the challenge strain. Hemagglutination inhibition (HI) titers in hens vaccinated with single- or double-volume doses are presented for each day post-challenge. The *X*-axis displays time in days; −7 indicates the time point corresponding to 14 days post-vaccination (dpv); 0 corresponds to 21 dpv; and 13 and 14 indicate days post-challenge. The horizontal dashed line indicates the detection limit (2 log_2_). The asterisk indicates a statistically significant difference (*p* < 0.05). (**B**) Kaplan–Meier survival curves illustrating the survival of laying hens after the challenge under the two vaccination regimens and the unvaccinated control group. Differences between vaccinated and unvaccinated groups were determined using the log-rank test (*p* < 0.05).

Given the potential emergency application, all poultry—including juvenile and adult chickens—were considered targets for vaccine administration, and protective immunity after a single-dose regimen was required. Two independent experimental rounds were conducted in hens to evaluate the efficacy of a single-dose versus a double-volume dose (Figure S7), assuming that a single dose might be insufficient to induce protective immunity in hens with larger body sizes than juvenile chickens. Consistent humoral immune responses were observed across both experimental rounds. Antibodies were induced in all vaccinated hen groups, and HI titers remained comparable between 14 and 21 dpv and between single- and double-volume regimens (*p* > 0.05) (Figure 5A). At 14 dpc, HI titers increased in surviving hens, with the magnitude of increase varying according to clinical outcome; GMTs were significantly higher than before challenge (*p* < 0.05). Although the HI titers at challenge were similar, the protective efficacy differed between regimens (Figure 5B). The double-volume regimen provided greater protection, with only one hen exhibiting mild clinical signs, including depression, ruffled feathers, loss of appetite, and cessation of egg production from 5 dpc. Conversely, the single-dose regimen provided partial protection in both rounds and was associated with consistent clinical signs. In round 1, three of the four vaccinated hens developed clinical signs beginning at 4 dpc, initially presenting with transient prominent periorbital and facial swelling and progressing to severe neurological signs, including torticollis or coma. One hen was euthanized at 13 dpc after reaching the humane endpoint. Similarly, limited efficacy was observed in round 2, where two of the four hens died at 5 and 7 dpc, with symptoms of fatigue and progressive lethargy. Although egg production continued before clinical deterioration, eggshells were thin and fragile on the last day of production (3 and 5 dpc). Of the remaining two surviving individuals, one remained asymptomatic, whereas the other showed neurological signs with mild neck torticollis and ceased egg production starting at 7 dpc.

**Figure 6:**
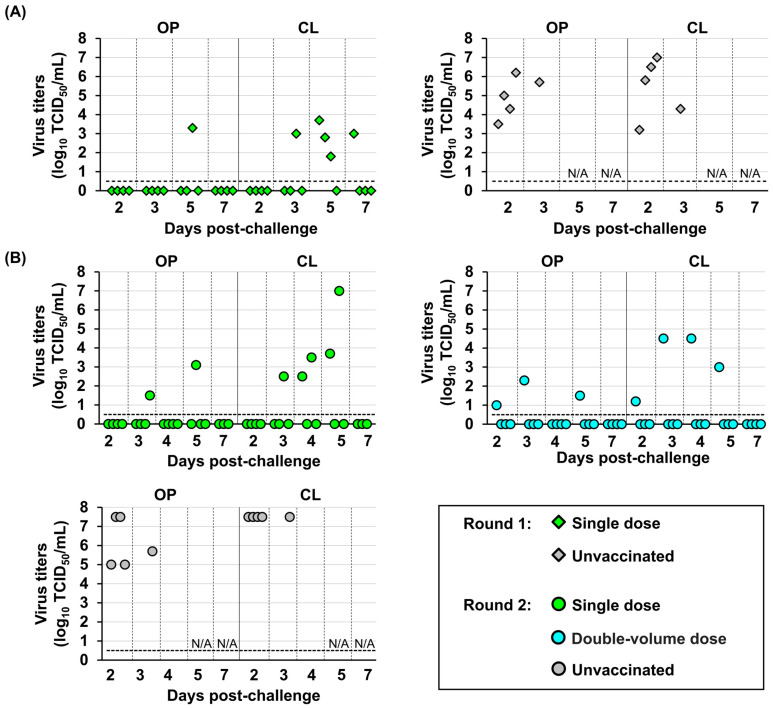
Virus recovery from oropharyngeal (OP) and cloacal (CL) swabs of laying hens following challenge under different vaccination regimens in rounds 1 (**A**) and 2 (**B**). Virus titers are expressed as log_10_ of the 50% tissue culture infectious dose (TCID_50_/mL). The horizontal dashed line indicates the detection limit (0.5 log_10_ TCID_50_/mL). N/A: not available due to chicken mortality.

High viral titers were detected in both oropharyngeal and cloacal swabs from all unvaccinated hens (Figure 6). In the single-dose group, viruses were recovered from three of 4 vaccinated chickens in round 1 and from all vaccinated chickens in round 2. Compared with the unvaccinated groups, viral shedding was delayed in some individuals, with titers peaking at 4–5 dpc and persisting up to 7 dpc. Conversely, in the double-volume regimen group, virus recovery was limited to a single hen that displayed mild clinical signs. This hen showed virus recovery at 2–5 dpc, with higher titers in cloacal swabs, peaking at 4.5 log_10_ TCID_50_ at 3 and 4 dpc and reaching the lowest titer of 1.2 log_10_ TCID_50_ at 2 dpc.

**Figure 7:**
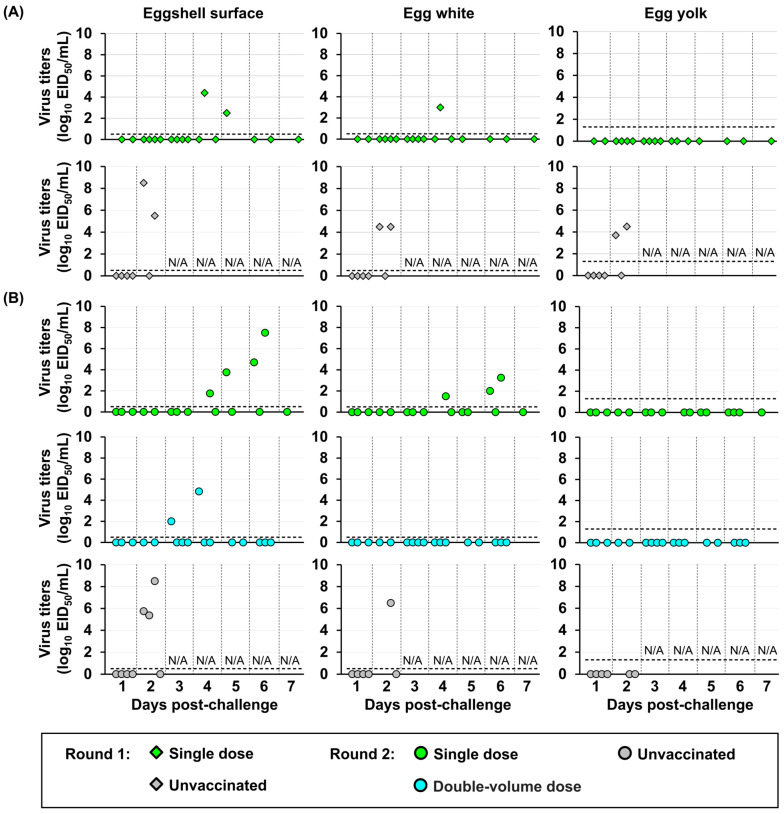
Viral contamination in egg components (eggshell surface, egg white, and egg yolk) of laying hens following challenge with single- or double-volume dose vaccines in two independent experiment rounds, including rounds 1 (**A**) and 2 (**B**). Virus titers are expressed as log_10_ of the 50% egg infectious dose (EID_50_). The horizontal dashed line indicates the detection limit (0.5 log_10_ EID_50_/mL for eggshell surface and egg white and 1.3 log_10_ EID_50_/mL for egg yolk). N/A: not available due to chicken mortality. At 7 dpc, egg samples were rarely obtained because most hens did not lay eggs on that day.

Virus contamination in egg products was also evaluated (Figure 7). Eggs laid by hens in the single-dose vaccine group exhibited higher viral contamination than those laid by hens in the double-volume regimen group. In the single-dose vaccine group, the infectious virus was detected not only on the eggshell surface but also within the egg white, peaking at 3.3 log_10_ EID_50_ at 6 dpc and reaching the lowest titer of 1.5 log_10_ EID_50_ at 4 dpc. Conversely, in the double-volume dose group, the infectious virus was detected only on the eggshell surface of eggs laid by hens exhibiting viral shedding in cloacal swabs, with titers of 2.0–4.8 log_10_ EID_50_ between 3 and 4 dpc. Notably, no detectable virus was confirmed in the egg yolk of eggs from any vaccinated chickens. Conversely, viruses were detected in the egg white and egg yolk and on the eggshell surface of eggs laid by unvaccinated hens.

## 4. Discussion

In this study, a new candidate vaccine derived from a representative field strain was established. Its protective efficacy was evaluated in comparison with an antigenically homologous vaccine to the challenge strain and a Japanese stockpiled vaccine. Multiple trials were also conducted to determine the earliest time point at which protection could be achieved after vaccination and to assess vaccine efficacy in laying hens, aiming to improve field control strategies for HPAI. The findings underscore differential protective outcomes depending on both antigenic relatedness and host age. First, under the degree of genetic and antigenic divergence evaluated, the stockpiled vaccine completely protected juvenile chickens against mortality, although limited and transient viral shedding was observed. Second, despite antigenic matching between vaccine and challenge strains, vaccinated laying hens were not fully protected against morbidity and mortality, even when HI antibody titers against the challenge strain reached the required protective levels. These results suggest that although a certain degree of antigenic divergence may still permit protection against mortality in juvenile chickens, optimal and consistent protection requires antigenic matching. Importantly, the low efficacy observed in laying hens despite antigenic compatibility further indicates that effective emergency vaccination depends on appropriate strain selection and on vaccination strategies capable of ensuring sufficient protection in adult birds.

Interestingly, although the antigenic distance among AIVs is generally thought to increase proportionally with their genetic distance [21], a divergence from this pattern was observed in this study. Specifically, although clade 2.3.4, 2.3.4.4e, 2.3.4.4g, and 2.3.4.4h viruses were antigenically distant from classic viruses, the circulating clade 2.3.4.4b viruses appeared to revert antigenically toward the classic group despite accumulating further genetic distance. These findings suggest that the viruses were selected under immune pressures favoring antigenic structures similar to the classic group rather than simply exhibiting novel antigenic properties. This phenomenon may be explained by the fact that genetic changes in the HA gene do not uniformly affect key epitopes involved in immune recognition. Antigenicity can depend on a limited number of key amino acid residues near the receptor binding site [22]. Amino acid position 158 on HA may represent a key residue, as it can serve as a potential glycosylation site that obscures antigenic epitopes and allows viruses to evade antibody recognition. However, none of the clade 2.3.4.4b viruses used in this study possessed the glycosylation site within the 158–160 region. Therefore, the efficacy of available or candidate vaccines against field viruses, identified through routine monitoring, should be evaluated based on both genetic and antigenic analyses rather than genetic analysis alone.

According to the WOAH requirements, HI titers must be at least 32 (5 log_2_) to confer protection from mortality or more than 128 (7 log_2_) to reduce recovery of the challenge virus [2]. Although the HI titers of some vaccinated juvenile chickens immediately before challenge were lower than these thresholds, all vaccinated juvenile chickens survived for 14 days after HPAIV challenge. A high level of near-sterile protection was confirmed in both the rgPR8/VN23 (H5N1) and NIID-002 (H5N1) vaccine groups, with no detectable virus shedding in 9 of 10 chickens. This robust protection, despite suboptimal antibody levels immediately before challenge, may be correlated with ongoing immunity development, as the antibody response had not yet peaked at 21 dpv. In a previous study, the antibody titers induced by stockpiled vaccine peaked at 6–7 weeks post-vaccination and remained high for extended periods [6]. Infectious viruses were detectable in the 4 chickens vaccinated with the Dk/Hok/Vac-1/04 (H5N1) vaccine, suggesting partial protection resulting from an antigenic gap between the vaccine and challenge strains. Although antigenic matching is critical for vaccine efficacy, NA-specific immunity can also contribute to protection against HPAIV infection. NA promotes the release of newly formed virions by hydrolyzing sialylated glycans, and antibodies against NA can limit this process [23]. Although NA was not genetically and antigenically analyzed in this study, the origins of NA in the vaccine strains allow for the consideration of its potential contribution to protection. rgPR8/VN23 (H5N1) and NIID-002 (H5N1) vaccine strains both contain NA derived from representative HPAIV field strains, whereas the Dk/Hok/Vac-1/04 (H5N1) vaccine strain possesses NA from a non-pathogenic AIV [6]. These different NA origins may cause minor genetic and antigenic differences. Based on NA sequence information, Dk/Hok/Vac-1/04 (H5N1) and Fox/Hok/1/22 (H5N1) strains shared similar amino acids located in the NA active site or the conserved epitope at positions 222–230 [23]. This conservation suggests that NA antibodies may have contributed to the reduced viral shedding and survival observed in chickens vaccinated with Dk/Hok/Vac-1/04 (H5N1). In addition, the degree of protection can vary depending on the NA subtype. A previous study reported that chickens vaccinated with an H5N1 inactivated vaccine survived challenges with heterologous H5Nx HPAIVs within clade 2.3.4.4, including H5N2, H5N6, and H5N8, although viral shedding occurred in vaccinated chickens challenged with H5N6 and H5N8 but not with H5N2 [24]. Therefore, both HA antigenic matching and NA-specific immunity likely influenced vaccine protection, including the reduction in viral shedding.

The early-protection experiment demonstrated that a protective effect of the vaccine against mortality was observable as early as 8 dpv, with one of the four chickens surviving despite the absence of detectable antibodies immediately before challenge. This finding is consistent with a previous study in Hong Kong in which vaccination was implemented as an outbreak response, showing that vaccinated chickens experienced reduced mortality 9–18 days after vaccination, even though HI antibody titers had not fully developed [25]. These observations support the notion that inactivated H5 vaccines can confer early clinical protection before peak humoral responses. Although cell-mediated and innate immune responses were not evaluated in this study, the protection prior to detectable HI antibody responses may represent emerging immunity during the early phase of immune development. Furthermore, complete suppression of viral shedding in vaccinated chickens is required to prevent virus spread within flocks, mitigating the risk of antigenic variant generation through continuous circulation. The infectious dose of AIVs varies significantly across bird species. Previous studies have reported that at least 3.4 log_10_ EID_50_ viruses are required to infect chickens, whereas fewer than 1.0 log_10_ EID_50_ viruses can infect ducks [26]. These results highlight the concern that HPAIV circulation at low titers promotes the emergence of antigenic variants through continuous infections in flocks highly sensitive to HPAIV. Therefore, effective post-vaccination surveillance strategies are indispensable in countries employing routine immunization programs.

Independent experiments in vaccinating laying hens revealed limitations of the standard single-dose regimen. Although the antibody titers immediately before challenge met the WOAH requirement of 32 HI (5 log_2_), a single dose was insufficient to fully protect the hens in this study. This finding aligns with a previous finding in hens, where a single dose induced a mean titer of 45 HI but failed to provide complete protection against virus contamination in egg products despite antigenic matching between vaccine and challenge strains [27]. Additionally, antibody kinetics after vaccination differed between juvenile chickens and laying hens in this study. Whereas juvenile chickens showed a significant increase in HI titers between 2 and 3 weeks post-vaccination, hens exhibited a comparatively stable antibody profile during the same interval, suggesting age-related differences in vaccine-induced humoral responses. Consistent with a previous study, antibody levels in vaccinated 76-week-old chickens were similar at 3 and 8 weeks post-vaccination, whereas a marked increase in antibodies was observed in vaccinated 4-week-old specific pathogen-free chickens [28]. The lower vaccine efficacy observed in 40-week-old hens compared with 4-week-old chickens may be related to the immune system’s development and function, particularly B cells that differentiate in the bursa of Fabricius. In hens, the bursa of Fabricius undergoes physiological involution beginning approximately at 10–16 weeks of age and is nearly complete at 24 weeks, resulting in a reduced capacity for the generation of new B cells [29,30]. Although data on B-cell activity, lymphocyte counts, and innate immunity were not collected in the present study, these physiological factors provide a plausible explanation for the lower vaccine efficacy observed in the 40-week-old hens. Previous studies have often focused on booster regimens to achieve high antibody titers and protection [27,31], which may limit rapid implementation during emergency vaccination. In this study, a single-dose regimen consistent with emergency-use policy was applied to evaluate vaccine efficacy under conditions where booster administration is not required. Relative vaccine dosage per body weight may influence the serological response in birds, as supported by a previous study in zoo birds [32]. Thus, the true effective dose administered to hens should be reconsidered relative to that used in juvenile chickens. To test this hypothesis, a double-volume dose was administered to hens. Notably, the double-volume dose did not significantly alter HI antibody titers compared with the single dose. However, the double-volume dose group demonstrated improved clinical protection and substantially reduced viral shedding and virus contamination in egg products. In a previous study, booster vaccination achieved the HI levels required for reducing virus recovery (GMT of 330 ± 101), yet virus contamination was still detected in all egg contents [31]. In contrast, the present study observed an effective reduction in virus contamination in egg white and yolk with only 5 log_2_ (32 HI). The virus was detected only on the eggshell surface of eggs from one hen, likely due to virus shedding in the cloacal tract. These findings indicate that HI titers alone may not fully reflect vaccine-mediated protective effects of inactivated vaccines. Although HI titers are widely used as a correlate of protective efficacy, they primarily reflect the ability of humoral immunity to inhibit hemagglutinin-mediated viral attachment. Given that mechanistic immune responses were beyond the scope of the present study, the immunological basis of this observation should be determined. Collectively, these findings indicate that even an antigenically matched vaccine cannot fully confer protective immunity to adult laying hens under a single-dose regimen. HPAI vaccination policies vary between countries that permit routine vaccination and those that restrict vaccines to emergency use only. The optimized single-dose approach evaluated in this study provides practical insights for improving vaccine efficacy under urgent field conditions, representing an advantage relative to previous reports [27,31]. Consequently, the limited protective efficacy observed in 40-week-old laying hens after a single dose reflects the practical limitations of the emergency-use framework in field settings. Given these constraints, vaccination-based control strategies should prioritize optimizing the timing—preferably administering vaccines before 16 weeks of age—for routine use in countries that permit vaccination. In countries adopting an emergency-only vaccination policy, including Japan, a relative vaccine dosage according to body weight should be considered.

RT-qPCR is a highly sensitive tool for identifying H5 HPAIV in disease-free areas or during early outbreak stages. However, under vaccination or endemic settings, interpreting positive RT-qPCR results becomes more complex. In this study, positive Ct values were obtained from samples of vaccinated chickens, although infectious viruses were not recovered from the same samples. This discrepancy arises because viral RNA may persist long after virus clearance following vaccination or recovery, leading to positive PCR results even in protected animals. Consequently, positive PCR results may lead to misinterpretation of infection status. Differentiating vaccinated, infected, and uninfected individuals based solely on Ct values remains challenging, given the principles of gene-detection diagnostics. Therefore, in post-vaccination monitoring contexts, positive qPCR results must be interpreted cautiously, and virus isolation remains the gold standard for confirming active infectious virus shedding and accurately assessing vaccine efficacy in the field.

## 5. Conclusions

The trial antigenically matched inactivated vaccine protected juvenile chickens after a single dose but did not fully protect laying hens against circulating H5N1 HPAIV belonging to clade 2.3.4.4b. Although the antigenically mismatched stockpiled vaccine prevented mortality in juvenile chickens, complete suppression of viral shedding occurred only with antigenic matching, underscoring its importance for optimal protection. In laying hens, the vaccination regimen influenced protective outcomes independently of measured HI titers. These findings highlight major limitations of inactivated vaccines and emphasize the need for continuous surveillance, genetic and antigenic analyses, post-vaccination monitoring, and optimized vaccination strategies, particularly for single-dose emergency use in adult birds.

## Data Availability

The original contributions presented in this study are included in the article. Further inquiries can be directed to the corresponding author.

## Supplementary Materials

The following supporting information can be downloaded at: https://www.mdpi.com/article/10.3390/vaccines14040291/s1, Figure S1: Growth kinetics of vaccine strains in embryonated chicken eggs; Figure S2: Growth stability of rgPR8/VN23HA∆KRRK-NA (H5N1) (A) and NIID-002 (A/Ezo red fox/Hokkaido/1/2022) (H5N1) (B) vaccine strains after five serial passages in embryonated chicken eggs (*n* = 3 eggs per passage); Figure S3: Schematic of vaccination, challenge, and sample collection for the evaluation of vaccine protective capacity in juvenile chickens; Figure S4: Antibody responses elicited by the rgPR8/VN23HA∆KRRK-NA (rgPR8/VN23; H5N1) (A) and A/duck/Hokkaido/Vac-1/2004 (Dk/Hok/Vac-1/04; H5N1) (B) vaccines against their homologous antigens, and comparison of hemagglutination inhibition (HI) titers against homologous and challenge strains (C); Figure S5: Virus recovery and M gene detection in a subset of swab samples from unvaccinated chickens (A) and chickens vaccinated with the rgPR8/VN23HA∆KRRK-NA (H5N1) (B) and A/duck/Hokkaido/Vac-1/2004 (H5N1) (C) vaccines; Figure S6: Survival and viral shedding of vaccinated chickens challenged at various days post-vaccination (dpv); Figure S7: Schematic of vaccination, challenge, and sample collection for the evaluation of vaccine protective capacity in the laying hens; Table S1: Primer and probe sequences used for reverse transcription quantitative polymerase chain reaction targeting the M gene of avian influenza virus; Table S2: Avian influenza surveillance in Vietnam from 2019 to 2024; Table S3: List of H5 avian influenza viruses used in genetic and antigenic analyses; Table S4: Cross-reactivity of H5 avian influenza viruses with antisera using hemagglutination inhibition assay; Table S5: The intravenous pathogenicity index of the vaccine strains; Table S6: Genetic stability of recombinant vaccine strains following five serial passages, comparing passages 1 and 5.

## Abbreviations

The following abbreviations are used in this manuscript:

AIVs	avian influenza viruses
BSL-3	biosafety Level 3
CLD_50_	50% chicken lethal dose
cRBCs	chicken red blood cells
dpc	days post-challenge
dpv	days post-vaccination
EID_50_	50% egg infectious doses
GISAID	Global Initiative on Sharing All Influenza Data
GMT	geometric mean titer
HA	hemagglutination/hemagglutinin
HEK293T	human embryonic kidney 293T
HI	hemagglutinin inhibition
HPAI	high pathogenicity avian influenza
HPAIV	high pathogenicity avian influenza virus
IVPI	intravenous pathogenicity index
MDCK	Madin–Darby canine kidney
MEM	minimum essential medium
PBS	phosphate-buffered saline
NA	neuraminidase
RT-qPCR	reverse transcription quantitative polymerase chain reaction
TCID_50_	50% tissue culture infectious dose
WOAH	World Organisation of Animal Health
