# Supplementary material for: Efficacy and Limitations of an Improved Vaccine Derived from an Updated Vaccine Strain Against H5 High Pathogenicity Avian Influenza
Source: Vaccines (Basel). 2026 Mar 24;14(4):291. doi: 10.3390/vaccines14040291 (PMC13120588; doi:10.3390/vaccines14040291)
Supplement: Supplementary file 1 [file vaccines-14-00291-s001.zip › vaccines-4094015-supplementary.pdf]

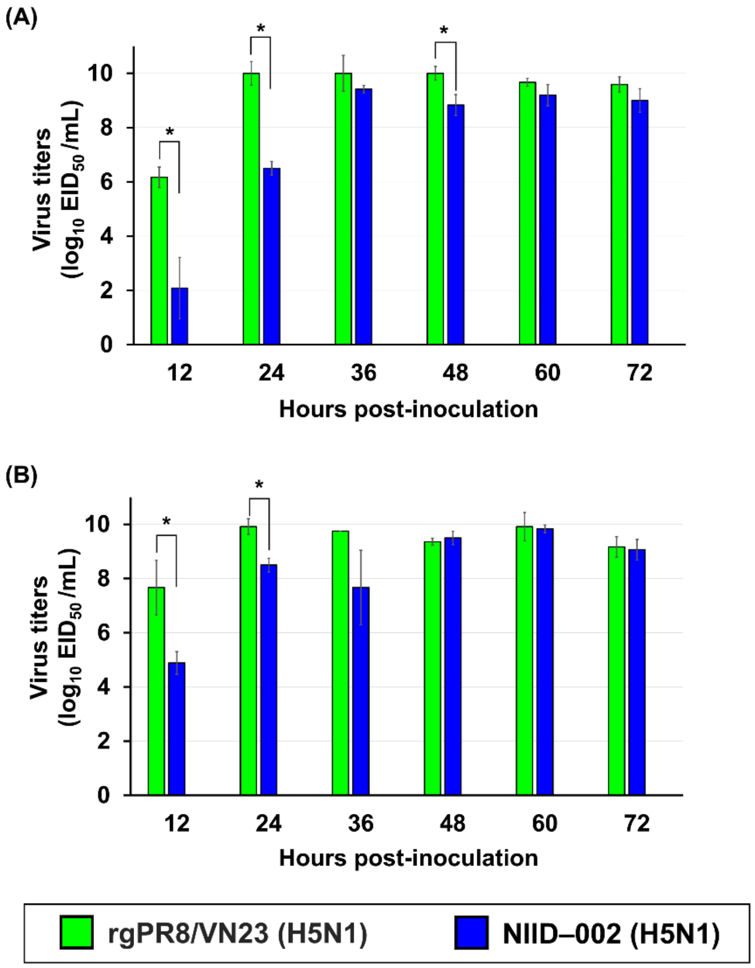

Figure S1. Growth kinetics of vaccine strains in embryonated chicken eggs. Vaccine strains were inoculated into eggs with $10^{2.0}$ **(A)** and $10^{4.0}$ 50% embryo infectious dose (EID_{50}) **(B)**. The colors represent different vaccine strains: green for the rgPR8/VN23HAΔKRRK-NA (rgPR8/VN23; H5N1) and blue for the NIID-002 (A/Ezo red fox/Hokkaido/1/2022) (NIID-002; H5N1). Virus titers are expressed as log_{10} of the EID_{50} and data represent the mean ± standard deviation. Statistical comparisons were performed using Student's *t*-test or one-way analysis of variance (ANOVA). The asterisk indicates a statistically significant difference ($p < 0.05$). All experiments were performed in triplicate (n = 3 eggs per time point).

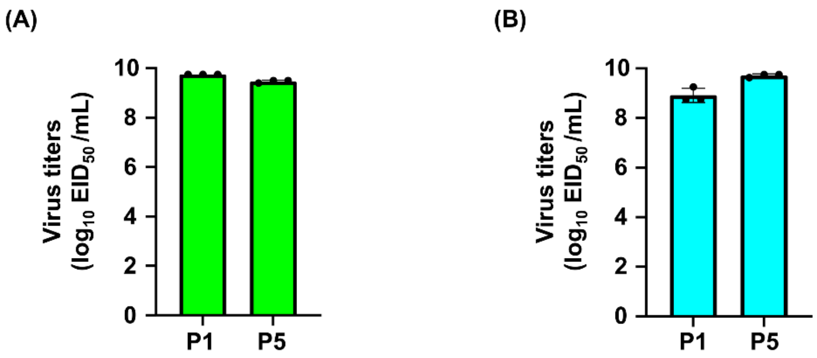

Figure S2. Growth stability of rgPR8/VN23HAΔKRRK-NA (H5N1) **(A)** and NIID-002 (A/Ezo red fox/Hokkaido/1/2022) (H5N1) **(B)** vaccine strains after five serial passages in embryonated chicken eggs (n = 3 eggs per passage). Viral titers were determined at passages 1 (P1) and 5 (P5). Virus titers are expressed as log_{10} of the 50% egg infectious dose (EID_{50}). Data are shown as median ± interquartile range. Statistical analysis was performed using the Wilcoxon signed-rank test, and no significant differences were observed.

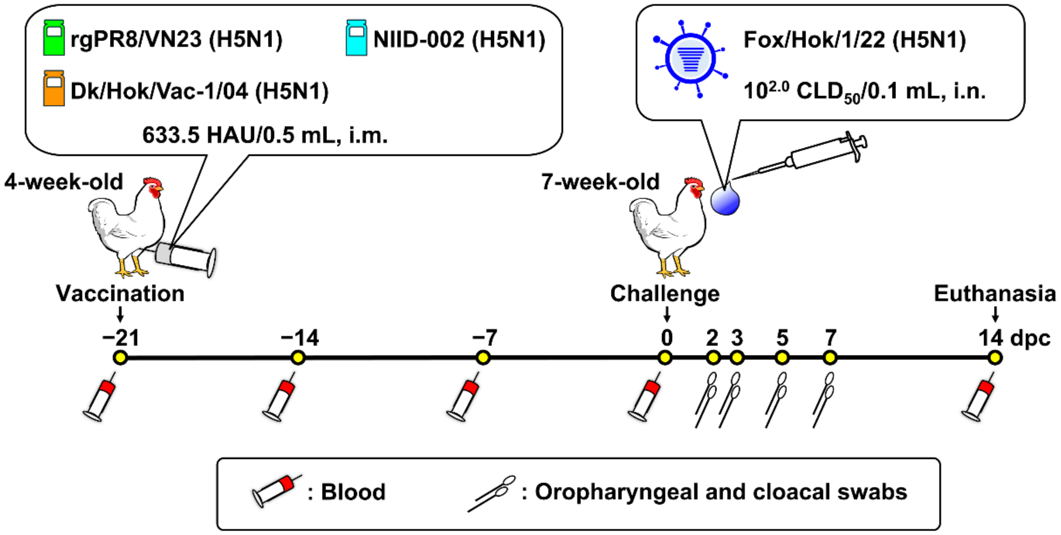

Figure S3. Schematic of vaccination, challenge, and sample collection for the evaluation of vaccine protective capacity in juvenile chickens. Four-week-old White Leghorn chickens were intramuscularly (i.m.) vaccinated with the rgPR8/VN23HAΔKRRK-NA (rgPR8/VN23; H5N1), NIID-002 (A/Ezo red fox/Hokkaido/1/2022) (NIID-002; H5N1), or A/duck/Hokkaido/Vac-1/2004 (Dk/Hok/Vac-1/04; H5N1) vaccines. Blood samples were collected before vaccination and at 7, 14, and 21 days post-vaccination (dpv), corresponding to −21, −14, and −7 days post-challenge (dpc), as well as at 14 dpc to measure the hemagglutination inhibition titers. At 21 dpv, chickens were intranasally (i.n.) challenged with $10^{2.0}$ 50% chicken lethal dose (CLD_{50}) of A/Ezo red fox/Hokkaido/1/2022 (Fox/Hok/1/22; H5N1). Oropharyngeal and cloacal swabs were collected at 2, 3, 5, and 7 dpc to assess the viral shedding.

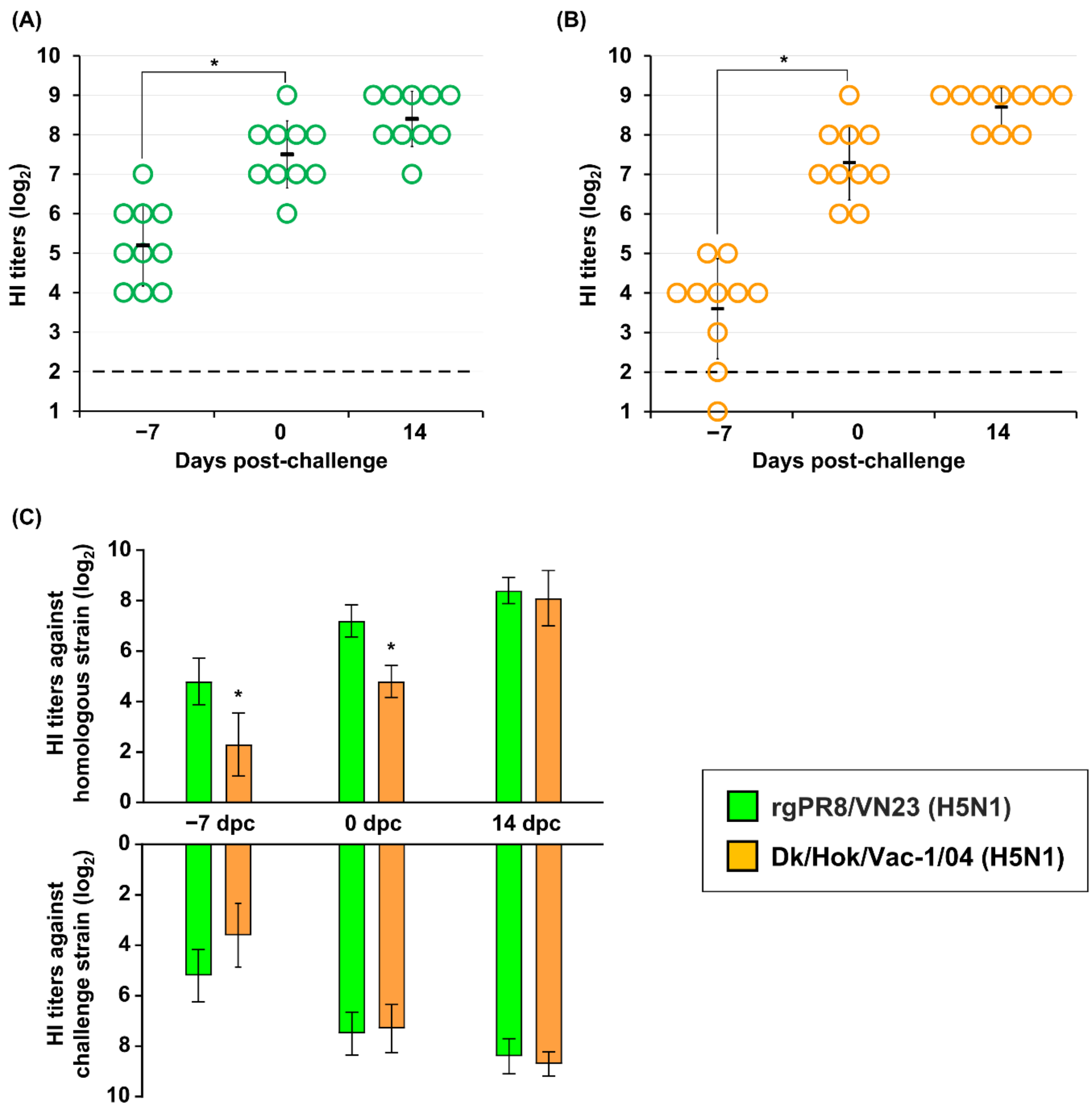

Figure S4. Antibody responses elicited by the rgPR8/VN23HAΔKRRK-NA (rgPR8/VN23; H5N1) **(A)** and A/duck/Hokkaido/Vac-1/2004 (Dk/Hok/Vac-1/04; H5N1) **(B)** vaccines against their homologous antigens, and comparison of hemagglutination inhibition (HI) titers against homologous and challenge strains **(C)**. The X-axis displays time in days; −7 indicates the time point corresponding to 14 days post-vaccination (dpv); 0 corresponds to 21 dpv; and 14 indicates 14 days post-challenge. The bars represent the geometric mean titers. Error bars represent the standard deviation of the log_2-transformed HI values. The asterisk indicates a statistically significant difference ($p < 0.05$). In panel A and B, individual HI titers are shown as open circles and the horizontal dashed line represents the detection limit (2 log_2). In the panel C, mean HI titers against homologous strains are shown above, and titers against the challenge strain are below in the bar charts, and the asterisk indicates a statistically significant difference between HI titers against homologous and challenge strains ($p < 0.05$).

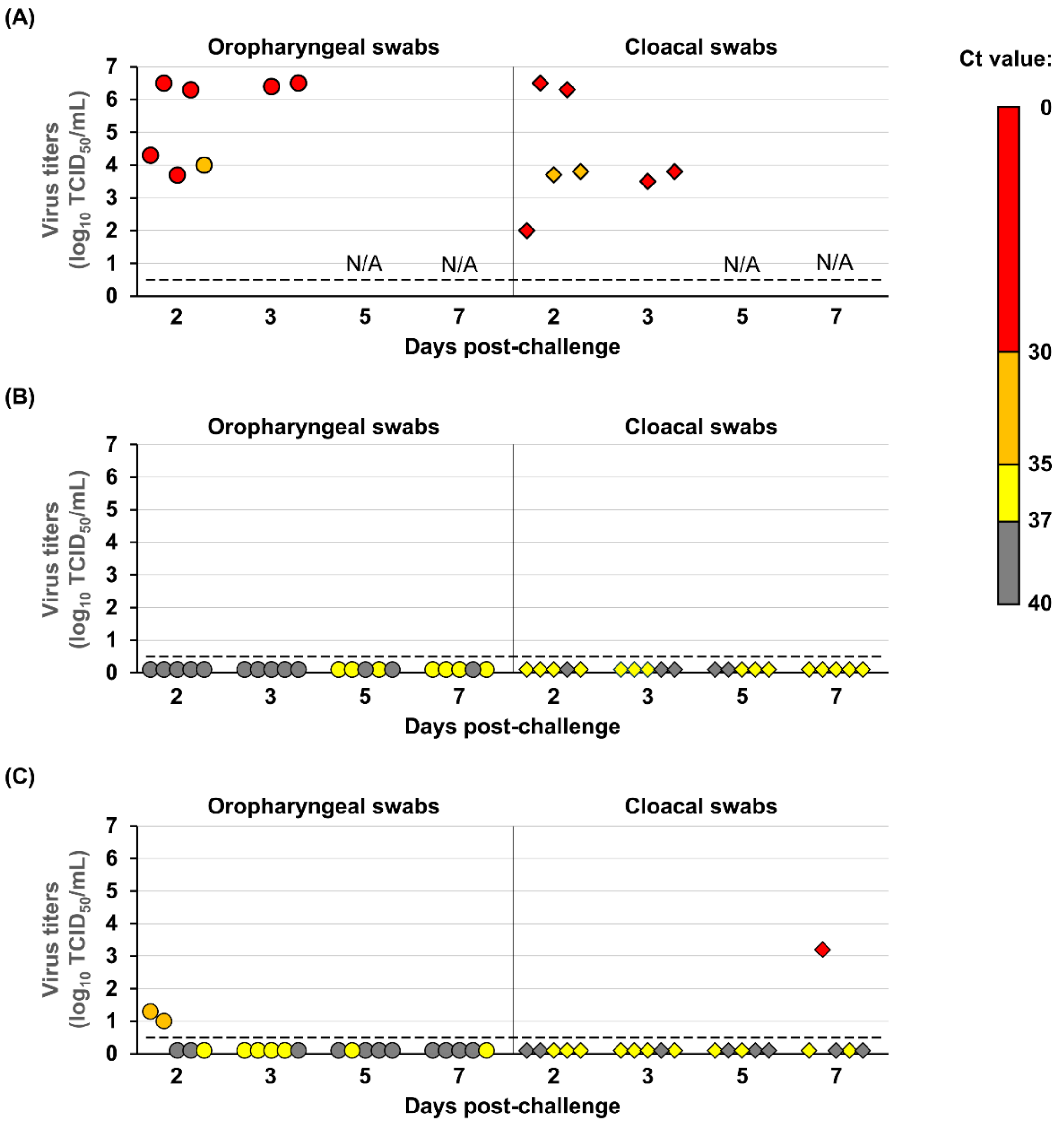

Figure S5. Virus recovery and M gene detection in a subset of swab samples from unvaccinated chickens **(A)** and chickens vaccinated with the rgPR8/VN23HAΔKRRK-NA (H5N1) **(B)** and A/duck/Hokkaido/Vac-1/2004 (H5N1) **(C)** vaccines**.** Infectious virus titers are plotted as individual circles and expressed as $\log_{10}$ of the 50% tissue culture infectious dose ($TCID_{50}$). The horizontal dashed line indicates the detection limit (0.5 $\log_{10}$ $TCID_{50}$/mL). Circle color corresponds to the cycle threshold (Ct) value. Reverse transcription quantitative polymerase chain reaction analysis was interpreted as follows: Ct ≥ 37 was considered negative based on pre-challenge swab samples, and Ct < 37 was considered positive. The color scale visually represents the full range of Ct values. N/A: Not available due to chicken mortality.

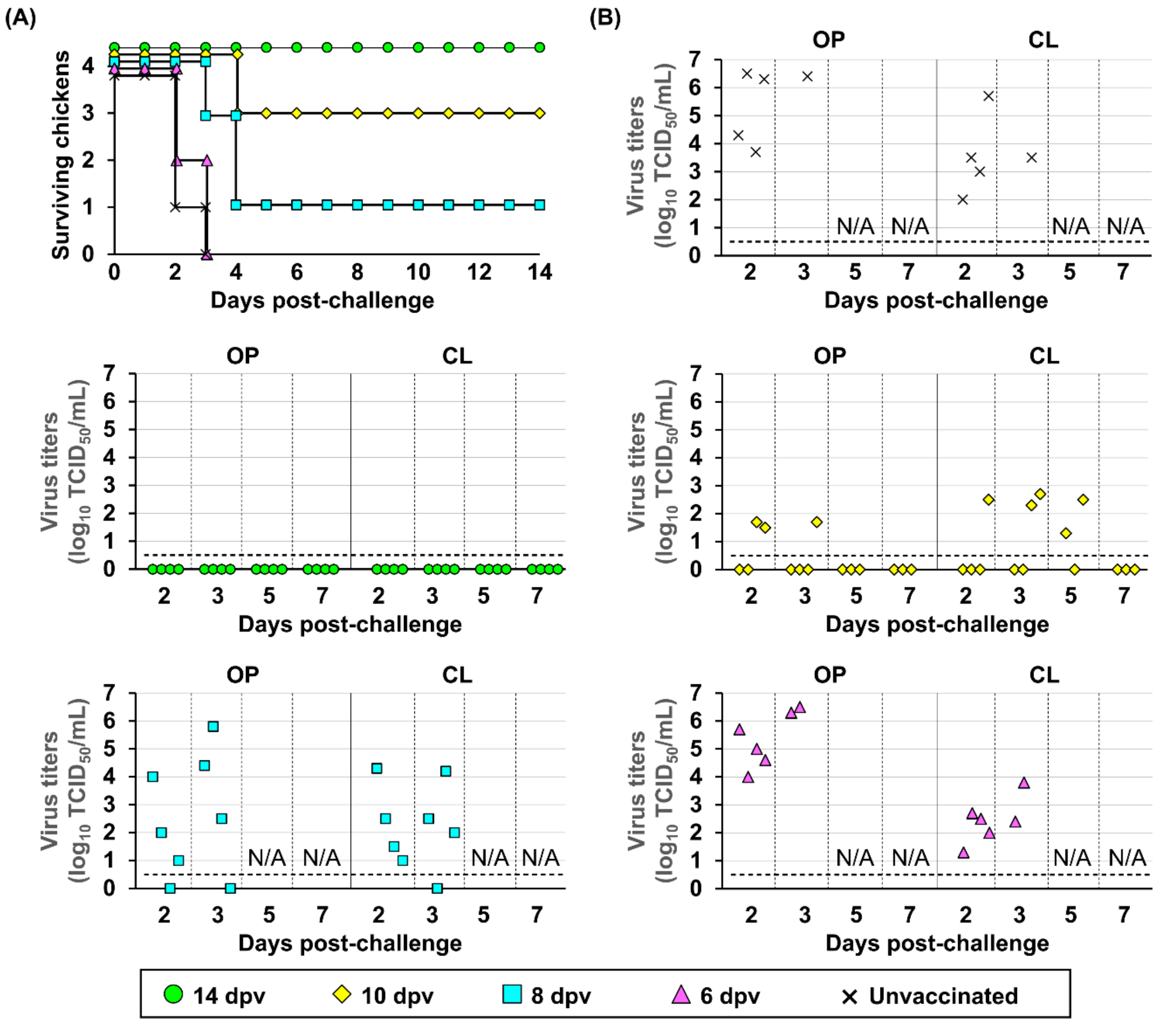

Figure S6. Survival and viral shedding of vaccinated chickens challenged at various days post-vaccination (dpv). **(A)** Survival curves were analyzed using the Kaplan–Meier method, and differences between groups were assessed using the log-rank test ($p < 0.05$). **(B)** Virus recovery from the oropharyngeal (OP) and cloacal (CL) swabs after the challenge. Viral titers are expressed as $\log_{10}$ of the 50% tissue culture infectious dose ($TCID_{50}$). The horizontal dashed line indicates the detection limit (0.5 $\log_{10}$ $TCID_{50}$/mL). N/A: Not available due to chicken mortality.

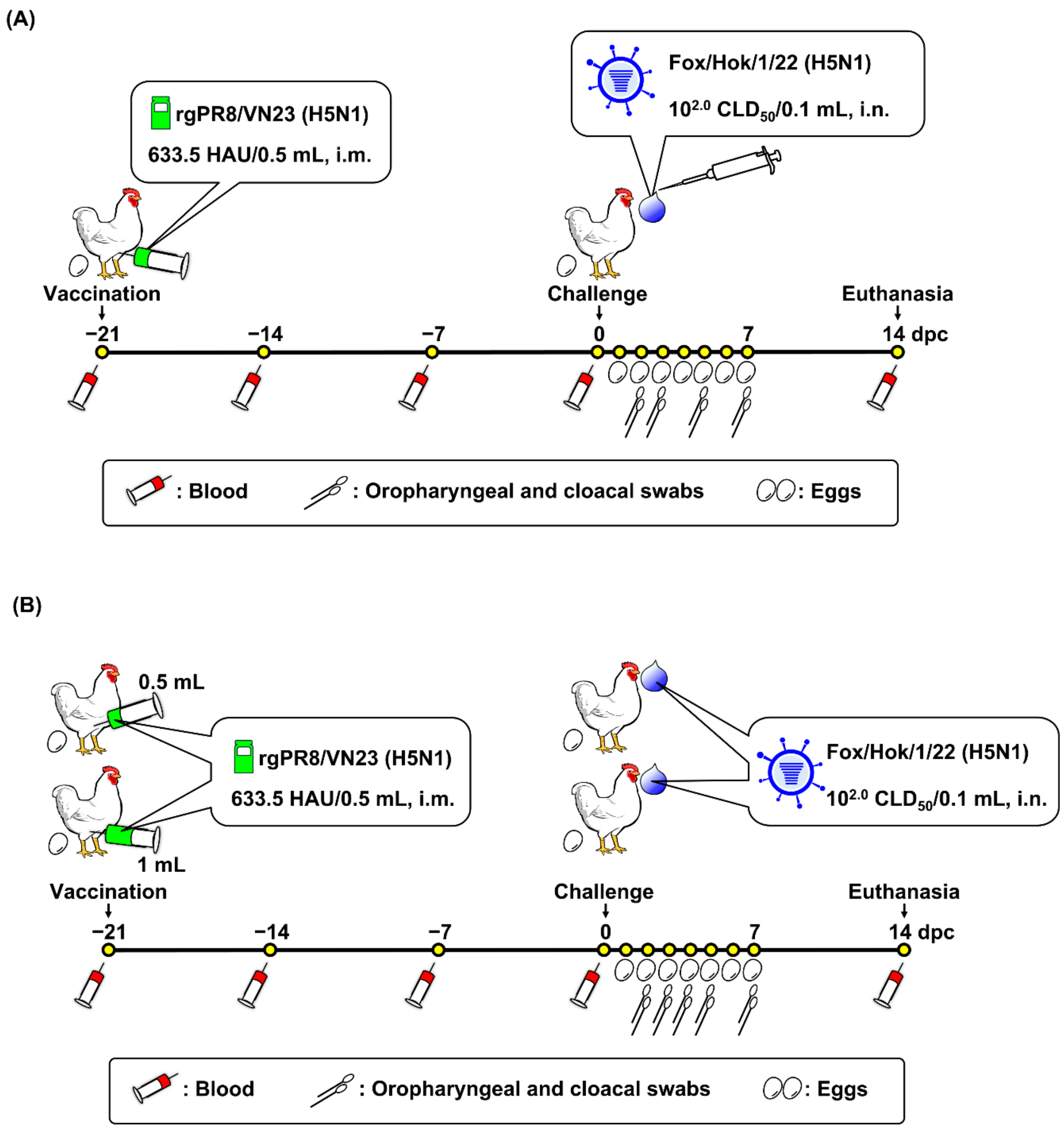

Figure S7. Schematic of vaccination, challenge, and sample collection for the evaluation of vaccine protective capacity in the laying hens. Forty-week-old White Leghorn chickens were intramuscularly (i.m.) vaccinated with rgPR8/VN23HAΔKRRK-NA (rgPR8/VN23; H5N1) vaccine. Blood samples were collected before vaccination and at 7, 14, and 21 days post-vaccination (dpv), corresponding to −21, −14, and −7 days post-challenge (dpc), as well as at 14 dpc to measure the hemagglutination inhibition titers. At 21 dpv, chickens were intranasally (i.n.) challenged with $10^{2.0}$ 50% chicken lethal dose (CLD_{50}) of A/Ezo red fox/Hokkaido/1/2022 (Fox/Hok/1/22; H5N1). Oropharyngeal (OP) and cloacal (CL) swabs were collected to assess the viral shedding, and eggs laid by hens were collected daily from 1–7 dpc to detect H5 HPAIV contamination. **(A)** Round 1: hens received a single-dose vaccination; OP and CL swabs were collected at 2, 3, 5, and 7 dpc. **(B)** Round 2: hens received either a single dose or a double-volume dose; OP and CL swabs were collected at 2, 3, 4, 5, and 7 dpc.

Table S1. Primer and probe sequences used for reverse transcription quantitative polymerase chain reaction targeting the M gene of avian influenza virus.

Name	Type	Sequence (5′–3′)*
D161M–F	Primer	AGATGAGYCTTCTAACCGAGGTCG
D162M–R1	Primer	TGCAAAAACATCYTCAAGTCTCTG
D162M–R2	Primer	TGCAAACACATCYTCAAGTCTCTG
D162M–R3	Primer	TGCAAAGACATCYTCAAGTCTCTG
D162M–R4	Primer	TGCAAATACATCYTCAAGTCTCTG
IVA MA	Probe	FAM–TCAGGCCCCCTCAAAGCCGA–TAMRA

*FAM: 5’-end fluorescent reporter; TAMRA: 3’-end quencher dye.

Table S2. Avian influenza surveillance in Vietnam from 2019–2024.

Year	Region	Province	No. of samples	AIV*-positive samples	Subtypes (no. of isolates)
2019	South	Vinh Long	1,634	109	H5N1 (12); H5N6 (22); H6N6 (31); H9N2 (42); H10N3 (2)
		An Giang	200	77	H5N6 (9); H9N2 (51); H6N6 (14); H3N2 (3)
	North	Lang Son	1,000	206	H5N6 (2); H6N6 (25), H9N2 (179)
2021	South	Vinh Long	800	84	H9N2 (32); H6N6 (48); H3N8 (3); H6N2 (1)
	North	Lang Son	800	150	H5N6 (4); H5N8 (21); H9N2 (64); H9N4 (1); H6N6 (38); H6N4 (1); H6N2 (1); H6N3 (2); H3N2 (12); H3N4 (5); H3N8 (1)
2023	North	Lang Son	800	80	H5N1 (24); H6N6 (19); H9N2 (26); H3N2 (11)
2024	North	Lang Son	800	43	H5N1 (14); H9N2 (24); H6N6 (5)

*AIV: avian influenza virus.

Table S3. List of H5 avian influenza viruses used in genetic and antigenic analysis.

Virus name[1]	Subtype	Clade	Abbreviation[2]	Accession No.
**A/duck/Vietnam/HU17-DD44/2024**	H5N1	2.3.4.4b	—	EPI_ISL_19385878
**A/duck/Vietnam/HU17-DD99/2024**	H5N1	2.3.4.4b	—	EPI_ISL_19386159
**A/duck/Vietnam/HU16-DD3/2023**	H5N1	2.3.4.4b	Dk/VN/DD3/23	EPI_ISL_18241793
**A/duck/Vietnam/HU16-DD121/2023**	H5N1	2.3.4.4b	—	EPI_ISL_18241794
**A/duck/Vietnam/HU16-DD125/2023**	H5N1	2.3.4.4b	—	EPI_ISL_18241795
**A/chicken/Vietnam/HU16-DD139/2023**	H5N1	2.3.4.4b	—	EPI_ISL_18241796
**A/Muscovy duck/Vietnam/HU16-DD144/2023**	H5N1	2.3.4.4b	—	EPI_ISL_18241797
**A/duck/Vietnam/HU16-NS82/2023**	H5N1	2.3.4.4b	Dk/VN/NS82/23	EPI_ISL_18241798
A/Eurasian wigeon/Hokkaido/Q71/2022	H5N1	2.3.4.4b	EW/Hok/Q71/22	EPI_ISL_15576617
A/white-tailed eagle/Hokkaido/22-RU-WTE-2/2022	H5N1	2.3.4.4b	WTE/Hok/R22/22	EPI_ISL_11330431
A/Ezo red fox/Hokkaido/1/2022	H5N1	2.3.4.4b	Fox/Hok/1/22	EPI_ISL_12174842
**A/Muscovy duck/Vietnam/HU14-GV50/2021**	H5N8	2.3.4.4b	Mdk/VN/GV50/21	EPI_ISL_20075820
**A/duck/Vietnam/HU14-GV60/2021**	H5N8	2.3.4.4b	—	EPI_ISL_20075822
**A/duck/Vietnam/HU14-GV125/2021**	H5N8	2.3.4.4b	—	EPI_ISL_20075824
**A/chicken/Vietnam/HU14-LB11/2021**	H5N8	2.3.4.4b	—	EPI_ISL_11504587
**A/Muscovy duck/Vietnam/HU14-LB18/2021**	H5N8	2.3.4.4b	—	EPI_ISL_20075818
A/northern pintail/Hokkaido/M13/2020	H5N1	2.3.4.4b	Np/Hok/M13/20	EPI_ISL_697771
**A/chicken/Vietnam/HU11-903/2019**	H5N6	2.3.4.4h	Ck/VN/903/19	EPI_ISL_20075827
**A/duck/Vietnam/HU13-65/2019**	H5N6	2.3.4.4h	—	EPI_ISL_503609
**A/duck/Vietnam/HU12-971/2019**	H5N6	2.3.4.4g	Dk/VN/971/19	EPI_ISL_503600
A/Muscovy duck/Vietnam/HU7-20/2017	H5N8	2.3.4.4g	Mdk/VN/20/17	EPI_ISL_295819
A/black swan/Akita/1/2016	H5N8	2.3.4.4e	Bs/Aki/1/16	EPI_ISL_243058
A/chicken/Kumamoto/1-7/2014	H5N8	2.3.4.4c	Ck/Kum/1-7/14	EPI_ISL_159719
**A/chicken/Vietnam/HU12-657/2019**	H5N1	2.3.2.1e	Ck/VN/657/19	EPI_ISL_504982
A/duck/Vietnam/HU3-836/2015	H5N6	2.3.2.1e	Dk/VN/386/15	EPI_ISL_17768752
A/chicken/Vietnam/HU4-42/2015	H5N8	2.3.4.4	Ck/VN/42/15	EPI_ISL_293989
A/peregrine falcon/Hong Kong/810/2009	H5N1	2.3.4	Pfal/HK/810/09	EPI_ISL_62730
A/Muscovy duck/Vietnam/OIE-559/2011	H5N6	1.1	Mdk/VN/559/11	EPI_ISL_91622
A/duck/Hokkaido/Vac-1/2004	H5N1	Classic	Dk/Hok/Vac-1/04	EPI_ISL_356

[1]Isolates from this study are indicated in bold. Isolates selected for antigenic analysis are underlined.
[2]Abbreviations are provided only for isolates included in the antigenic analysis.

Table S4. Cross-reactivity of H5 avian influenza viruses with antisera by hemagglutination inhibition assay.

Virus[1]	Clade	Antisera[2,3]								
		Dk/VN/ DD3/23	EW/Hok/ Q71/22	WTE/Hok/ R22/22	Dk/VN/ 20/17	Bs/Aki/ 1/16	Ck/Kum/ 1-7/14	Pfal/HK/ 810/09	Dk/VN/ 386/15	Dk/Hok/ Vac-1/04
**Dk/VN/DD3/23**	2.3.4.4b	640	80	640	40	80	320	80	160	40
**Dk/VN/NS82/23**	2.3.4.4b	320	40	160	20	80	40	<20	80	40
EW/Hok/Q71/22	2.3.4.4b	640	320	160	160	160	320	80	160	80
WTE/Hok/R22/22	2.3.4.4b	640	160	320	80	80	640	20	160	40
Fox/Hok/1/22	2.3.4.4b	640	160	640	80	160	320	<20	160	40
**Mdk/VN/GV50/21**	2.3.4.4b	320	160	1280	80	80	640	20	160	20
Np/Hok/M13/20	2.3.4.4b	640	20	160	160	40	640	20	160	20
**Ck/VN/903/19**	2.3.4.4h	<20	<20	<20	20	80	<20	<20	20	<20
**Dk/VN/971/19**	2.3.4.4g	160	80	320	320	320	320	320	160	<20
Mdk/VN/20/17	2.3.4.4g	40	20	80	80	80	80	40	40	<20
Bs/Aki/1/16	2.3.4.4e	160	20	80	80	640	160	<20	40	20
Ck/Kum/1-7/14	2.3.4.4c	160	40	20	80	80	640	<20	160	20
Ck/VN/42/15	2.3.4.4	320	40	1280	640	80	320	320	160	<20
Pfal/HK/810/09	2.3.4	80	<20	40	80	40	<20	320	40	<20
**Ck/VN/657/19**	2.3.2.1e	<20	<20	80	<20	<20	80	40	640	80
Dk/VN/386/15	2.3.2.1e	<20	20	160	20	<20	160	<20	1280	80
Mdk/VN/559/11	1.1	40	<20	160	40	40	<20	20	160	40
Dk/Hok/Vac-1/04	Classic	20	20	40	20	40	<20	<20	20	640

[1]Abbreviation names are indicated in Table S3. Viruses from this study are bold.
[2]Homologous titers are underlined.
[3]Hemagglutination inhibition titers below the detection limit for the assay are indicated as <20.

Table S5. The intravenous pathogenicity index of the vaccine strains.

Group	Chicken ID	Score[1,2]										IVPI[3]
		1 dpc	2 dpc	3 dpc	4 dpc	5 dpc	6 dpc	7 dpc	8 dpc	9 dpc	10 dpc	
rgPR8/VN23HAΔKRRK-NA (H5N1)	006	0	0	0	0	0	0	0	0	0	0	0.0
	007	0	0	0	0	0	0	0	0	0	0	
	008	0	0	0	0	0	0	0	0	0	0	
	009	0	0	0	0	0	0	0	0	0	0	
	010	0	0	0	0	0	0	0	0	0	0	
	011	0	0	0	0	0	0	0	0	0	0	
	012	0	0	0	0	0	0	0	0	0	0	
	013	0	0	0	0	0	0	0	0	0	0	
NIID-002 (A/Ezo red fox/Hokkaido/1/2022) (H5N1)	016	0	0	0	0	0	0	0	0	0	0	0.0
	017	0	0	0	0	0	0	0	0	0	0	
	018	0	0	0	0	0	0	0	0	0	0	
	019	0	0	0	0	0	0	0	0	0	0	
	020	0	0	0	0	0	0	0	0	0	0	
	021	0	0	0	0	0	0	0	0	0	0	
	022	0	0	0	0	0	0	0	0	0	0	
	023	0	0	0	0	0	0	0	0	0	0	

[1]Scores were defined as follows: 0, normal with no signs; 1, sick with a single sign (respiratory symptom, depression, diarrhea, cyanosis, edema, or nervous symptom); 2, seriously sick with multiple signs; and 3, death.
[2]dpc: days post-challenge.
[3]IVPI: intravenous pathogenicity index.

Table S6: Genetic stability of recombinant vaccine strains following five serial passages, comparing passage 1 and passage 5.

Vaccine strain	Gene segment	Nucleotide mutation
rgPR8/VN23HA∆KRRK-NA (H5N1)	PB2	None
	PB1	None
	PA	None
	HA	None
	NP	None
	NA	None
	M	None
	NS	None
NIID-002 (A/Ezo red fox/Hokkaido/1/2022) (H5N1)	PB2	None
	PB1	None
	PA	None
	HA	None
	NP	None
	NA	None
	M	None
	NS	None
